# High-throughput design of catalytic materials for NO_x_ reduction from aircraft emissions

**DOI:** 10.1038/s41524-026-02220-9

**Published:** 2026-07-09

**Authors:** Anshuman Goswami, Frank Abild-Pedersen, John W. Lawson, Joakim Halldin Stenlid

**Affiliations:** 1 Department of Chemical Engineering, Stanford University, Stanford, CA USA; 2 SUNCAT Center for Interface Science and Catalysis, SLAC National Accelerator Laboratory, Menlo Park, CA USA; 3 KBR, Inc., Intelligent Systems Division, NASA Ames Research Center, Moffett Field, CA USA; 4 Intelligent Systems Division, NASA Ames Research Center, Moffett Field, CA USA; 5 Department of Chemistry and Chemical Engineering, Chalmers University of Technology, Gothenburg, Sweden

**Keywords:** Chemistry, Engineering, Materials science

## Abstract

NO_x_ emissions from aircraft exhausts are a major source of air pollutants. Catalytic converters have been proposed as potential technologies to reduce NO_x_ emissions but the high temperatures and rapid exhaust gas flows pose challenges to catalyst design. Ni-based alloys are routinely used for high-temperature applications and exhaust systems; however, standard alloys are insufficient to catalyze NO_x_ reduction effectively. To assess whether alloying Ni with elements active for NO_x_ chemistry can improve performance, we conduct a high-throughput screening study, leveraging Density Functional Theory (DFT) calculations across an expansive materials space. We complement this with microkinetic analyses to identify optimal descriptor spaces for catalyst design. We consider a series of Ni-based equi-compositional ternary alloys that include both temperature-resistant elements (Fe, Co, Cr) and catalytically active metals (Pt, Pd, Rh) for NO_x_ reduction. These alloys are then evaluated according to their catalytic reactivity, high-temperature stability, and coking resistance. Through this in-silico high-throughput screening framework, we identify Ni-Co-Pd, Ni-Co-Pt, Ni-Fe-Pt and Ni-Cr-Pt as promising candidates that satisfy the performance criteria.

## Introduction

Nitrogen oxide (NO_x_, x = 1,2) emissions from air traffic have a significant impact on global anthropogenic climate change^1,2^. NO_x_ is known to alter the atmospheric concentration of two important greenhouse gases – ozone (O_3_) and methane (CH_4_)^3–5^ – with an enhanced climate effect when emitted at high altitudes. In addition, NO_x_ affects local air quality and human health, both through emissions near airports and from emissions at cruising altitudes^6–8^. Growing concerns over NO_x_ have led to increasingly stringent emissions standards in recent years^1,9,10^. Although NO_x_ emissions can be reduced by altering the fuel-to-oxygen ratio in the jet engine, such changes would come at the cost of an undesirable increase in CO_2_ emissions. As an alternative, mitigation strategies such as the introduction of reducing agents like H_2_ or NH_3_ into the exhaust gas have been proposed. In this work, we evaluate the use of catalytic materials designed to transform NO_x_ in the exhaust gas into benign species before release. However, in contrast to automotive applications, aviation systems face significant technological challenges in implementing such solutions.

The concerted reduction of NO_x_ coupled to oxidation of CO and unburned hydrocarbons (H_y_C_z_), also known as Three-way catalysis, is a commercial technology in automotive exhausts^11,12^

1 $${{NO}}_{x}\left(g\right)+{xCO}\left(g\right)\to \frac{1}{2}{N}_{2}\left(g\right)+{{x}\,{CO}}_{2}\left(g\right)$$

2 $$\frac{1}{x}\left(2z+\frac{y}{2}\right){{NO}}_{x}\left(g\right)+{H}_{y}{C}_{z}\left(g\right)\to \frac{1}{2x}\left(2{\rm{z}}+\frac{y}{2}\right){N}_{2}\left(g\right)+z{{CO}_{2}}\left(g\right)+{\frac{y}{2}}{{H}_{2}}O\left(g\right)$$

Supported precious metal catalysts, particularly the platinum group metals (PGM) such as Pt, Pd, and Rh, have shown promise for the above chemistry^13–15^ and their demand in the automotive industry has steadily increased over the years^16^. However, applying these materials under aircraft exhaust conditions may not be feasible. Emissions from aircraft engines primarily consist of CO_2_, N_2_, H_2_O, O_2_, CO, NO_*x*_, hydrocarbons and soot^17,18^ and this resulting chemical environment can lead to poisoning of the active sites on PGMs due to corrosion, sulfidation or sintering. Operational temperatures for these materials are extremely high and can exceed 1000 K during turbojet operation^17^. Under such conditions, PGMs are susceptible to deactivation through mechanisms such as sintering, creep, and coking, leading to a loss of active sites and catalytic degradation^14,19^. Additionally, aircraft exhaust speeds can be very high, ranging from 670 to 1300 miles per hour^20^, with a laminar flow lowering the residence time of the NO_x_ near the catalyst surface, placing further demands on the activity and stability of the catalytic material.

Identifying suitable catalytic materials therefore necessitates exploration of materials that are: (1) resistant to high temperatures and creep, (2) resistant to coking, and (3) can optimally catalyze NO_x_ reduction at short contact times. Nickel and nickel-based alloys have been employed for aircraft turbines and aerospace applications, due to their excellent physical properties at elevated temperatures^21^. Recently, a team at the Glenn Research Center at National Aeronautics Space Administration (NASA) developed high-temperature resistant Ni-based materials, alloyed with Cr/Co/Fe^22–24^. They have shown that the creep resistance properties are significantly improved upon addition of phase formers, allowing for access to higher operational temperatures^25^. While this strengthens high temperature properties, their catalytic properties and the possibility to improve upon them are presently unclear.

Theoretical studies based on Density Functional Theory (DFT) calculations are at the forefront for accelerating discovery of sustainable catalysts^26–28^. High-throughput computational design of catalytic materials has been explored for multiple reaction chemistries, including ammonia synthesis^29,30^, CO methanation^31^, dry reforming of methane^32^, and NO decomposition^33^. For transition metal surfaces, a paradigm for theory-aided catalyst exploration has been established^34,35^, which involves applying scaling relations^36^, Transition State Scaling (TSS) relationships^37,38^, and Sabatier analysis through microkinetic modeling^34^. This methodology, extensively applied to monometallic surfaces, has also found application for bimetallic surfaces^39–42^, with a recent contribution focusing on NO decomposition^40^. However, extension of these concepts to tri-metallic and mid-to-high entropy alloy candidates requires further exploration, with the indication of possible deviation from scaling relationships^28,43^_._ In addition, while prior methods focused exclusively on optimization of kinetic metrics including reaction rates and product selectivity, stability of the materials and susceptibility of the materials towards poisoning effects like coke formation deserve parallel emphasis in evaluating viability and long-term functioning of the catalyst. These aspects become paramount in the analysis of high-temperature applications, where durability of the catalyst is an important consideration alongside catalytic activity.

In this contribution, we present an in-silico high-throughput study of Ni-based alloys proposed to be active for NO_x_ reduction, focusing on NO (x = 1) conversion as the most significant NO_x_ gas in aircraft exhaust. We conduct a systematic adsorption study of O, N, C, NO, and CO adsorbates on fcc (111) and (100) terrace, and (211) stepped surface motifs of mid- to late transition metals within an expansive materials space and derive scaling relationships from this dataset. We further evaluate activation barriers of O_2_, N_2_, CO, NO, and CO_2_ dissociation on these surfaces and derive facet-specific TSS relationships for these steps. Through descriptor-based microkinetic analyses, we identify design spaces optimal for NO reduction in exhaust conditions. We subsequently consider Ni-based equi-compositional alloys, including thermally stabilizing metals (Fe, Cr, Co) and metals active for NO_x_ reduction (Pd, Pt, Rh). We compute descriptors for the above reaction and subsequently estimate thermal stability, kinetic performance and coking tendency of these alloys. Through this in-silico high-throughput screening framework, we identify Ni-Co-Pd, Ni-Co-Pt, Ni-Fe-Pt and Ni-Cr-Pt as plausible candidates for high temperature catalytic converters due to their predicted stability at high temperatures, resistance to coking, and high activity for the NO_x_ reduction.

## Results

### Linear scaling relationships

Our chosen reaction mechanism (Eqs. (15)–(21)) comprises five adsorbates (O, C, N, NO, and CO). We evaluated their adsorption energies on all binding sites on fcc (100), (111), and (211) surfaces. A schematic of all the surfaces and considered adsorption sites is shown in Supplementary Fig. S2. Supplementary Tables S1–S3 report the preferred binding sites for every adsorbate-metal system on the (111), (100) and (211) surfaces, respectively. On the (111) surfaces, a consistent preference for 3-fold fcc/hcp sites is observed for the atomic species. For the molecular species, no consistent preference for binding site is seen. These findings agree well with prior literature investigating atomic and molecular adsorption on multiple (111) surfaces^44–52^. On the (100) surfaces, C prefers 4-fold hollow sites consistently. N also mostly binds on 4-fold hollow sites with Pt(100) being the sole exception. O interestingly prefers binding on 4-fold hollow sites for the 3*d* and 4*d* transition metals and changes to bridge sites for the 5*d* transition metals. For CO and NO, again, no consistent preference is seen for the binding sites. On the (211) surfaces, C prefers the five-fold hollow sites, except for Au, Pt, Ir, and Os, which are 5*d* metals. O and N do not show any inclination for a particular site. CO and NO are more inclined to bind on step locations for all metals, with Ni being an exception. Results for the (100) and (211) surfaces reconcile with prior literature studies that screened multiple surfaces for CO oxidation and NO oxidation^33,53^.

**Fig. 1:**
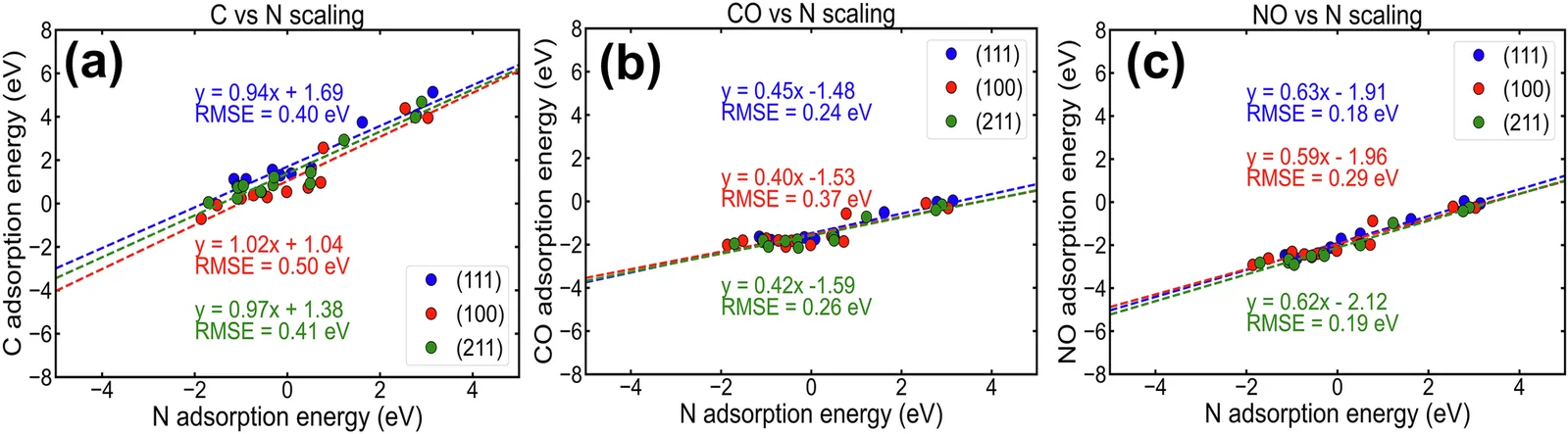
Linear adsorption-energy scaling relationships for reaction intermediates with N adsorption. In (**a**), C vs N, (**b**) CO vs N, and (**c**) NO vs N. Scaling relations are given for on fcc(111), fcc(100) and fcc(211) surfaces of the Ag, Au, Co, Cu, Ir, Ni, Os, Pd, Pt, Re, Rh, Ru, and Os transition metals. Data points correspond to the adsorption energies on the most preferred sites. Scaling relationships and associated root mean squared errors, derived through linear regression, are indicated in each figure.

To enable screening over multiple transition metals, we establish linear scaling relationships between adsorption energies of surface species. This approach has been employed extensively in the literature^36,54,55^ and plays a crucial role to reduce the dimensionality of the screening task to a handful of binding energies (“descriptors”) that characterize a transition metal surface. Here, we implement the N and O adsorption energies as the descriptors, identical to the approach by Falsig et al. for NO decomposition^33^. We next investigated the existence of scaling relations between C vs N, CO vs N and NO vs N for the considered metal systems. Figure 1 reports those relations across the three crystalline surfaces chosen. These relations were regressed on the binding energies of the most favorable adsorption sites for all adsorbate-metal combinations.

In a scaling relation between any two adsorbates A and B,

3 $${E}_{B}=\gamma {E}_{A}+\xi$$

Where γ and ξ represent the slope and intercept of the relations. Figure 1a reports the scaling relations for C vs N. The slopes of scaling relations are approximately unity for all considered motifs. An appreciable scatter is observed in these fits with root-mean-squared errors up to 0.5 eV. Figure 1b reports scaling relations for CO vs N. Slopes within 0.5–0.6 are observed for all surfaces, consistent with prior observation^55^. Fitting errors are within 0.2–0.3 eV, which is within acceptable DFT uncertainty estimates. Figure 1c reports scaling relations for NO vs N. The slopes are again close to 0.5, in agreement with previous findings^55^. Fitting errors are acceptable, within 0.2–0.3 eV. The above correlations permit an acceptable reduction in materials space dimensionality and enable fast screening of potential catalyst candidates.

### Transition state scaling relationships

We next describe the activation barriers of the elementary steps (Eqs. (15)–(21)). We assume that the activation barrier of NO adsorption (Eq. (15)) or CO adsorption (Eq. (16)) is equal to the binding energy if it is positive, or zero otherwise. The remaining steps in the mechanism can be described directly or indirectly through dissociative chemisorption step of a gas-phase molecule (${AB}(g)+2* \,\leftrightarrow \,{A}^{* }+{B}^{* }$). The transition state energies, *E*_TS_, and reaction energies, Δ*E*, for these steps are defined as follows:

4 $${E}_{{TS}}={E}_{{TS}/{slab}}-{E}_{{slab}}-{E}_{g}$$

5 $$\Delta E={E}_{A/{slab}}+{E}_{B/{slab}}-{2E}_{{slab}}-{E}_{g}$$

Where ${E}_{x/{slab}},\,{E}_{{slab}}$ and ${E}_{g}$ represent the energies of adsorbate/transition state (x)-surface, clean surface and gas-phase systems, respectively. We assume that activation barriers are linearly correlated with their corresponding reaction energies:

6 $${E}_{{TS}}=\alpha{\Delta}E+\beta$$

Equation (6) is also known as a Transition State Scaling (TSS) Relationship. The slope α tracks the response in transition state energy with the reaction energy and the intercept β signifies the surface independent intrinsic activation barrier. The origin of these relationships can be rationalized through a close resemblance of the transition state and final state geometries^37,38^, and thereby their mode of interaction with the surface will be similar.

The TSS relation (Eq. (6)) allows for determination of Sabatier volcano curves where the catalytic activity of a material is plotted as a function of a physical property of the catalyst that quantifies its ability to bond with reactants/intermediates or product species^37,56^. Knowledge of these relationships enables identification of materials that optimally bind reaction intermediates and exhibit maximal catalytic activity. Prior studies have determined these relationships for a wide class of reactions^37,57,58^. These include C-O, N-N, O-O, and N-O dissociation steps, which are present in our reaction mechanism for NO_x_ conversion.

In our work, we model dissociation steps on the different surface facets with the following assumptions:

- For fcc(111) surfaces, the dissociated fragments (${A}^{* },\,{B}^{* }$) occupy neighboring fcc hollow sites.
- For fcc(100) surfaces, the dissociated fragments (${A}^{* },\,{B}^{* }$) occupy neighboring four-fold hollow sites.
- For fcc(211) surfaces, one dissociated fragment (O, N, O, O, or CO) occupies the step location, while the other fragment (O, N, N, C, or O) occupies the four-fold hollow location for (O_2_, N_2_, NO, CO, or CO_2_) dissociation, respectively.

**Fig. 2:**
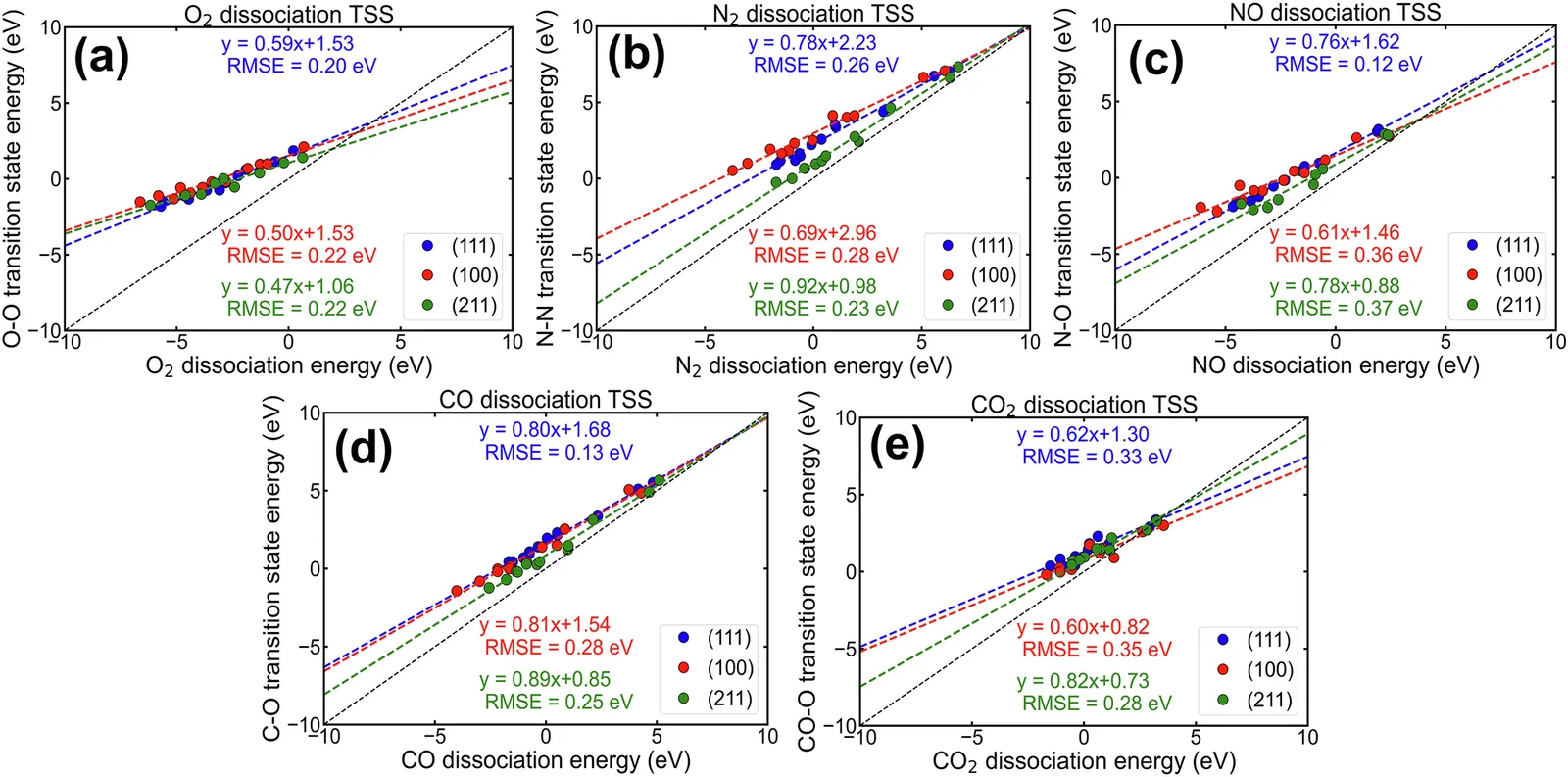
Transition-state scaling relationships (TSS) for O_2_, N_2_, NO, CO, and CO_2_ dissociation on monometallic fcc surfaces. Panels show relationships for (**a**) O_2_, (**b**) N_2_, (**c**) NO, (**d**) CO, and (**e**) CO_2_ dissociation on fcc(111), fcc(100), and fcc(211). The best-fit line and root mean squared error (RMSE) is reported for each relation. The black lines represent parity between transition state energies and dissociation energies.

Figure 2 depicts the TSS relationships for all dissociation systems with Supplementary Tables S4, S5, and S6 reporting the individual datapoints in each plot. The errors of fit range between 0.1 and 0.3 eV, comparable to DFT uncertainties within 0.2 eV. A remarkable feature for all dissociation reactions is the geometric similarity in the transition state structures, indicating bond breaking events are closely related for the considered systems.

The slopes for N_2_, CO, NO, and CO_2_ dissociation are all greater than 0.5 across the crystalline surfaces considered, indicating that the corresponding transition states are relatively final-state-like. The slopes of O_2_ dissociation agree, within uncertainty limits, with prior studies^33,53,58^. For all dissociation relationships except O_2_, the TSS lines for the fcc(111) surfaces are shifted upwards relative to the stepped (211) surfaces, with the fcc(100) surfaces lying in between. This follows the trend in surface coordination from fcc(211) via fcc(100) to fcc(111), indicating lower barriers (and increased reactivity) of increasingly undercoordinated sites.

### Microkinetic model

We now proceed to describe the activity of NO decomposition using the determined scaling and TSS relations. We make the following assumptions in our microkinetic model:

1. For consistency, we assume adsorbates exclusively occupy specific sites across all considered metals. These are the fcc-hollow, four-fold hollow, and a combination of step and five-fold hollow on fcc(111), fcc(100) and fcc(211) surfaces, respectively. These sites correspond to the actual site preferences in most of the cases, as seen from Supplementary Tables S1–S3. We further assume that the scaling relationships described in Fig. 1 would be representative of the site-specific binding energy correlations for each surface. We test these assumptions in Supplementary Fig. S3 and observe prediction errors approximately equal to the fits in Fig. 1.
2. We also neglect the formation of surface oxides during operation. This assumption would be valid for oxophobic transition metals like Pt/Pd/Rh. Despite the elevated temperatures, the formation of surface oxides may be possible under exhaust temperatures and would reduce the catalytic activity. Inclusion of their thermodynamics and reactivity is beyond the scope of the present study, but forms an important component for future in-silico design considerations.
3. Sites communicate with each other via surface diffusion. This precludes the effects from adsorbate-co-adsorbate interactions. This is a valid approximation due to high temperatures and high exhaust velocity conditions, which would reduce contact times and steady state coverage of weakly bound molecular adsorbates on the surface. Diffusion is also restricted to sites of the same types, e.g., between one fcc-hollow to another fcc-hollow site on an fcc(111) surface. This neglects spillover between different sites, the inclusion of which could improve model accuracy but would lead to an intractable number of site permutations and dissociations to consider.
4. Herein, adsorption energetics and dissociation barriers are evaluated in the low-coverage limit, enabling consistent comparison across a large set of alloy surfaces within a descriptor-based framework. This approximation neglects lateral adsorbate–adsorbate interactions, which may become important at moderate-to-high coverages and modify adsorption energies, reaction barriers, and absolute rates^59–61^. As a practical guideline, such effects are expected to become noticeable once surface populations reach ~0.25–0.50 ML^40,62,63^, although the precise onset depends on the adsorbate and surface structure. The approximation is therefore most reliable where adsorbate populations remain low, and less reliable in regions where O* or N* accumulate substantially. These effects are neglected here to retain computational tractability across the large alloy space considered, but their explicit inclusion would be a valuable direction for future refinement.

Using scaling relationships described in Section *Linear scaling relationships*, we can describe the binding energies of C, CO, and NO as a function of N and O binding energies, which are henceforth the descriptors of our reaction network. Together with the TSS relationships derived in Section *Transition State Scaling Relationships*, we can construct facet-dependent microkinetic models as a function of the descriptor-space, which span the materials space of interest.

**Fig. 3:**
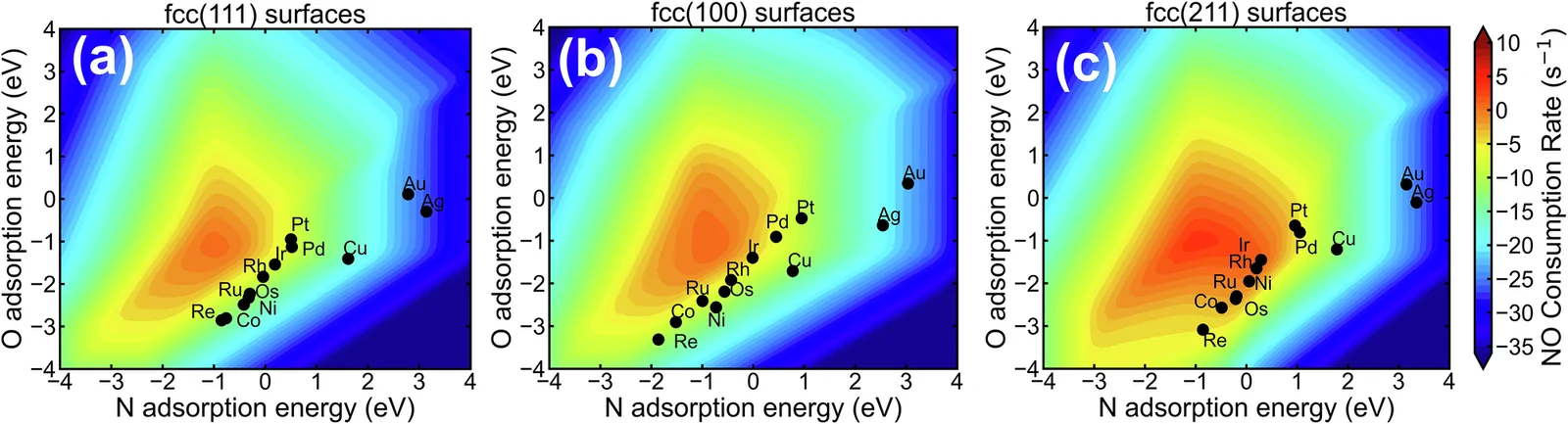
Mean-field microkinetic rates of NO decomposition as a function of O and N binding energies on fcc surfaces. Rates are evaluated on **a** fcc(111), **b** fcc(100), and **c** fcc(211) surfaces at *T* = 1100 K, *P*_N2_ = 0.7 bar, *P*_O2_ = 0.1 bar, *P*_CO_ = 0.0002 bar, *P*_NO_ = 0.002 bar and *P*_CO2_ = 0.04 bar. Data points correspond to adsorption energies on the considered elemental transition-metal surfaces.

We considered the aircraft turbine exhaust conditions as simulated in prior studies^64,65^ that reproduces measured exhaust gas compositions from standard jet engines^66,67^. The activity maps for NO decomposition rate are shown in Fig. 3. For the (111) and (100) surfaces, the data points correspond to adsorption energies on fcc hollow/4-fold hollow sites, respectively. For the (211) surfaces, the data points correspond to the mean adsorption energy over a step bridge and five-fold hollow location next to the step.

Consistent with Falsig et al.^33^, all maps are in the shape of a volcano with a specific region of descriptor space showing marked reactivity for this chemistry. The optimal regions are relatively widespread compared to Falsig et al.^33^, possibly due to higher temperatures considered (see Supplementary Figs. S7–S9 for maps at other temperature). Furthermore, as we progress from (111) to (100) to (211), the volcano shapes are increasingly widespread. This signifies that more metals, assuming stepped fcc(211) surfaces, can be active for the chemistry, as opposed to closed packed (111) and (100) surfaces. We consistently observe that Pt, Pd, and Rh for all three crystalline surfaces feature closer to the volcano top, compared to the other metals, agreeing with the prior knowledge of their enhanced activity in automotive exhausts^15^. Supplementary Fig. S4 depicts the activity maps for CO consumption rate for all three facets. These activity maps have similar descriptions as Fig. 3, with Pt, Pd, and Rh featuring close to the top of the volcano, in agreement with prior studies^53,68^.

We briefly comment on the error estimates in the microkinetic model predictions. These errors can originate from scaling relationships, which have fitting errors up to 0.5 eV (Fig. 1). In addition, the fitting errors in the transition state scaling relations (Fig. 2) are up to 0.4 eV. Assuming a slope of 0.9 in the transition state scaling, uncertainties in activation barriers can be up to 1.3 eV, leading to a maximum uncertainty in computed rate constants (and rates) of 6 orders of magnitude. This estimate should, however, be regarded as a conservative upper bound. In practice, the uncertainties are not strictly additive, as errors in related scaling relationships are often correlated, such that the effective uncertainty may be smaller than this worst-case estimate suggests. The above analysis agrees with Falsig et al.^33^, who observed deviations within 6 orders of magnitude in comparing rates from descriptor-based models versus a full microkinetic model across considered surfaces. Nonetheless, we also observe that the overall trends in NO decomposition rates are consistent with prior experimentally measured NO rates^69,70^, with those for Pt/Pd/Rh falling within 2 orders of magnitude of experiments. Moving forward, we use these analyses to identify broad trends and promising regions in descriptor space for alloy materials, as discussed in the next sections.

Coverage maps for the above models are shown in Supplementary Figs. S5, S6. We observe that N and O are the most abundant surface intermediates across much of the descriptor spaces. Consistent with Falsig et al.^33^, the considered transition metals fall primarily within the regions of high O surface coverage. We note, however, that the present microkinetic simulations do not explicitly include gas-phase transport or high-velocity flow effects. The predicted high coverages of O* and N* therefore reflect their thermodynamic stability and comparatively slow removal kinetics within the modeled reaction network. Accordingly, the descriptor maps identify regions of materials space that are predicted to become strongly O- or N-covered and thus less favorable for catalytic operation within the present framework. We note that the most reactive transition metals (Pt, Pd, and Rh) do not correspond to a high O coverage, which demonstrates their compromise between strong binding of intermediates and excellent activity for dissociation reactions. However, the application of pure Pt, Pd, and Rh based catalysts for high temperature NO_x_ conversion would not be feasible, owing to sintering tendencies, low thermal stability, and high costs^11,12^. We therefore turn our attention to alloys, which would be stable at high temperatures and be catalytically active under these conditions.

### Surface stability analysis of Ni-based alloys

We focus on the stability and reactivity of Ni-based alloys, which have a variety of aerospace applications^71–73^. Enhancing creep resistance is one of the enduring challenges in their long-term applications and increasing the upper limit of operational temperatures. Cr^74^, Co^75,76^ and Fe^75,77^ are among the candidates which have been studied for improving creep resistance and thermal stability of the alloy composites. Zacherl et al.^78^ highlighted through DFT calculations that the shear and Young’s modulus as well as the stacking fault energy of these alloys is proportional to the closeness of the additive relative to Ni. Wu et al. used DFT calculations to further demonstrate the promotional effects of these additives, due to a *d*-*d* hybridization that strengthens the Ni-X bond^79^. Recently, researchers at NASA demonstrated exceptional durability and creep life of a Ni-based superalloy composed of these additives, for turbine disks^22,23^. However, to the best of our knowledge, no studies have inspected the catalytic properties of these surfaces yet.

Building on the above insights, we investigate a materials space composed of Ni as the base element, Cr, Co, or Fe as the stabilizing elements and Pd, Pt, or Rh as the catalytically active elements. For comparison, we also evaluate ternary alloys without added Pd, Pt, and Rh. In total, we consider 12 material combinations: NiCrCo, NiCrFe, NiCoFe, NiCrPd, NiCrPt, NiCrRh, NiFePd, NiFePt, NiFeRh, NiCoPd, NiCoPt, and NiCoRh. We assume equal bulk compositions (33%) throughout. We investigate the fcc phase for the bulk alloys and fcc(111), (100), and (211) crystallographic motifs of the alloy surfaces, with the lattice constants set to the average of the individual elements in the fcc phase, following Vegard’s law^80^. We analyze the surface stability and use the descriptor-based analysis to estimate catalytic activity of these alloys for the remainder of the study.

For the bulk structures, we define a bulk formation energy as:

7 $${E}_{{A}_{x}{B}_{y}{C}_{z}}^{{bulk}}=\frac{{E}_{{A}_{x}{B}_{y}{C}_{z}}^{{DFT}}-x{E}_{A,{bulk}}^{{DFT}\,}-y{E}_{B,{bulk}}^{{DFT}\,}-z{E}_{C,{bulk}}^{{DFT}\,}}{x+y+z}$$

Where ${E}_{i,{bulk}}^{{DFT}}$ represents the formation energy per atom of each of each element in their most stable phase. This phase is preferred for all elements in the considered material space, except Cr and Fe that favor the body-centered cubic (bcc) phase. The preference for the fcc phase is further supported by a CALPHAD ternary stability analysis for the Ni-Co-Pt system based at 900 K, based on experimental unary and binary data (Supplementary Fig. S20)^81–83^. For each alloy, we construct a (3×3×3) fcc supercell comprised of 108 atoms and generate 10 plausible special quasi-random structures (SQS) using the capabilities of the Alloy Theoretical Automated Toolkit (ATAT)^84,85^. The alloy formation energy was computed and averaged over every structure. These alloy models are intended to represent idealized chemically disordered solid solutions for high-throughput screening of intrinsic catalytic trends. As such, they do not explicitly capture long-range ordering, clustering, phase separation, or other forms of microstructural evolution that may arise under synthesis or operating conditions and alter the population of active surface ensembles.

**Fig. 4:**
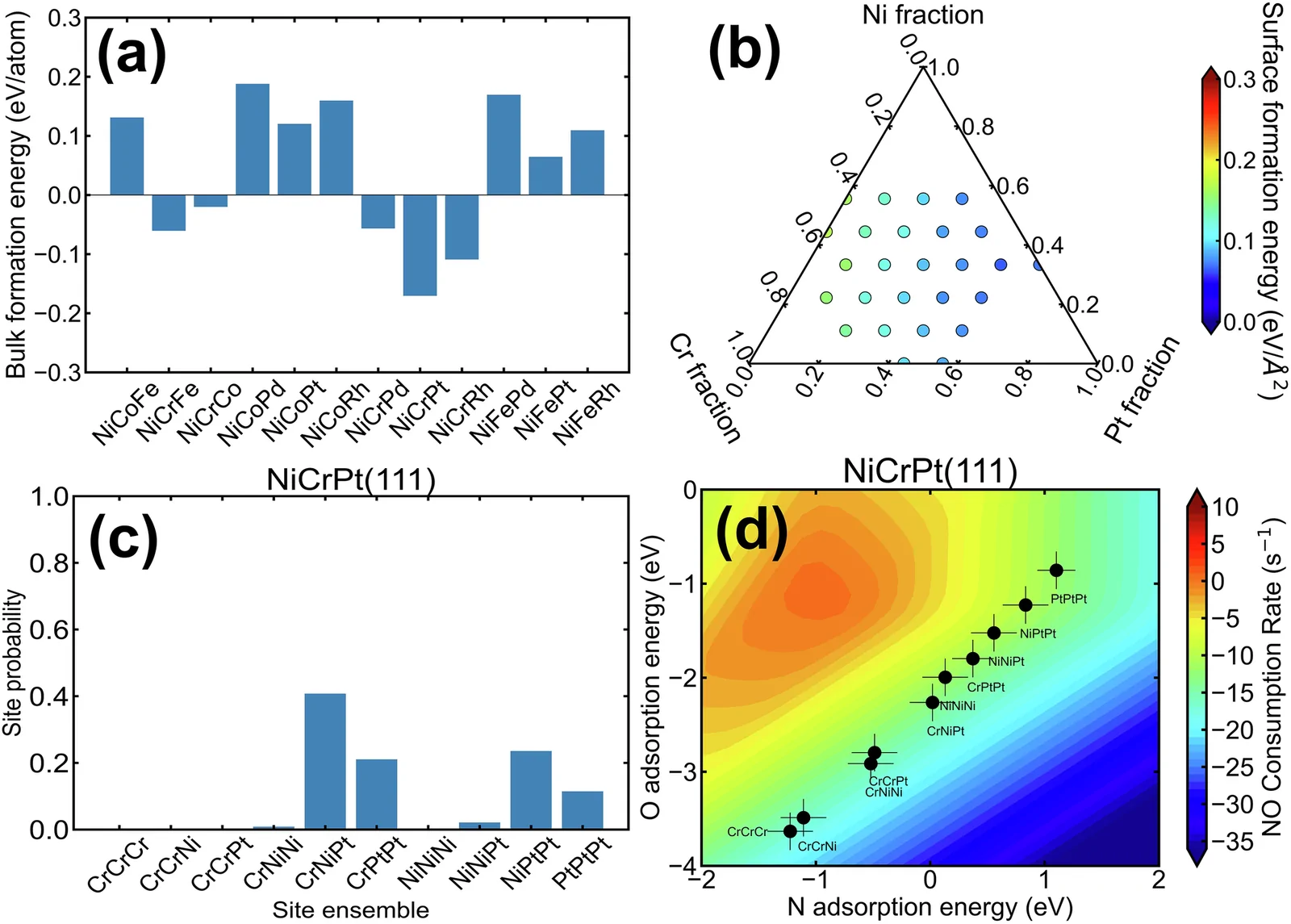
Bulk and surface energetics, site distributions, adsorption energetics, and predicted microkinetic activity of alloy surfaces. Panel (**a**) shows the bulk formation energies ${E}^{{bulk}}$ for all considered alloy surfaces. Panel (**b**) presents ternary diagram of NiCrPt(111) surface at T = 0 K indicating surface formation energy as a function of surface composition. Panel (**c**) shows site probability of fcc hollow sites on NiCrPt(111) at T = 1100 K. Panel (**d**) shows adsorption energies of site ensembles for NiCrPt(111) overlaid on the volcano plot for (111) surfaces. Error bars indicate corresponding standard deviations.

Figure 4a depicts the formation energies of the considered alloys. For the alloys without active elements, the addition of Cr appears to improve alloy stability considerably, followed by Fe and Co. The change in formation energy upon addition of active elements depends on the stabilizing element. For NiCo, alloying with Pt, Pd, or Rh leads to more endothermic formation energies relative to Cr and Fe. For NiFe, alloying with Pd (Pt, Rh) can increase (decrease) the formation energies relative to Cr and Co. For NiCr, alloying with Pt, Pd, and Rh causes more exothermic formation energies relative to Co and Fe. This observation is consistent with Wu et al.^79^, who found that Cr contributes to stability by reducing lattice mismatch. It is also seen that alloying with Pt leads to more exothermic formation energies, followed by Rh and lastly Pd. This trend agrees with Zacherl et al.^78^, who found that Pt and Rh contribute to increased creep resistance compared to Pd. The combination of NiCrPt has the most exothermic formation energy, drawing on the benefits of both alloying components.

We next analyzed the formation energy of alloy surfaces. The surface formation energy (normalized per unit cell area) of an ABC alloy is defined as follows:

8 $${{FE}}_{{A}_{x}{B}_{y}{C}_{z}}^{{surf}}=\frac{1}{{A}_{{cell}}}\frac{{E}_{{A}_{x}{B}_{y}{C}_{z}}^{{DFT},\,{surf}}-x{E}_{A,{bulk}}^{{DFT}\,}-y{E}_{B,{bulk}}^{{DFT}\,}-z{E}_{C,{bulk}}^{{DFT}\,}}{x+y+z}$$

Where ${E}_{i,{bulk}}^{{DFT}}$ represents the energy per atom of each element in their most stable phase (fcc for Ni, Pd, Pt, and Rh; hcp for Co; and bcc for Fe and Cr) and ${A}_{{cell}}$ is the area of the unit cell. We note that Eq. (8) assumes that the zero point and vibrational contributions are negligible compared the total bulk energy.

The temperature dependent free energy of the surface ${F}_{{A}_{x}{B}_{y}{C}_{z}}^{{surf}}\left(T\right)$ is expressed as:

9 $$\begin{array}{l}{F}_{{A}_{x}{B}_{y}{C}_{z}}^{{surf}}\left(T\right)={E}_{{A}_{x}{B}_{y}{C}_{z}}^{{DFT},\,{surf}}+{F}_{{A}_{x}{B}_{y}{C}_{z}}^{{vib}}\left(T\right)\,+\,{k}_{B}{T}\left[\right.{x}_{A,{surf}}\,{\mathrm{ln}}\,{x}_{A,{surf}}\\\qquad\qquad\qquad+\,{x}_{B,{surf}}\,{\mathrm{ln}}\,{x}_{B,{surf}}+{x}_{C,{surf}}\,{\mathrm{ln}}\,{x}_{C,{surf}}\left.\right]\end{array}$$

Where ${F}_{{A}_{x}{B}_{y}{C}_{z}}^{{vib}}\left(T\right)$ represents contributions from phonon vibrations and ${x}_{i,{surf}}$ represents the fraction of element i on the surface. The latter term in Eq. (9) effectively represents the ideal configurational entropy of mixing. Test calculations indicate that the phonon entropic contribution is constant (0.2–0.3 eV) across all tested alloy surfaces. For each alloy, the formation energy of over 200 inversion symmetric structures was considered for the fcc (100), (111), and (211) surfaces.

We comment here that the surface formation energies reported in Eq. (8) are evaluated for clean surfaces, providing a consistent baseline reference for comparing alloy stability across compositions. Under oxygen-covered conditions, adsorbed oxygen may alter surface energetics and promote adsorbate-induced segregation, particularly for more oxophilic elements, thereby changing the population of surface motifs relative to the clean-surface prediction. The clean-surface formation energies should therefore be interpreted as a baseline estimate rather than a full description of operando surface composition. A more rigorous assessment of oxygen-induced segregation would require explicit calculations in the presence of adsorbed oxygen, for example through atom-exchange or segregation-energy analyses, which is beyond the scope of the present study but represents an important direction for future work.

The slab models considered here are intended to represent idealized chemically mixed alloy environments and were not explicitly optimized with respect to segregation energies. The surface formation energies therefore provide a consistent baseline for comparing alloy compositions, but do not constitute a full prediction of equilibrium surface segregation. A more rigorous treatment of segregation-driven surface structures would require explicit evaluation of segregation energetics and configurational sampling across multiple slab compositions, which is beyond the scope of the present high-throughput screening study.

Figure 4b reports ${{FE}}_{{A}_{x}{B}_{y}{C}_{z}}^{{surf}}$ as a ternary diagram for NiCrPt (111) surface, illustrating the relationship between surface composition and formation energies. As the surface was modeled as a 3×3 unit-cell, the variations between surface compositions are discrete. We find that the surface becomes more stable with an increase in fraction of Ni and Pt atoms on the surface. This observation agrees with prior studies reporting segregation of Pt to the surface for a NiPt alloy^86,87^. Supplementary Figs. S10–12 collectively report ternary diagrams for all alloys in fcc(111), (100), and (211) motifs. In general, for alloys without active elements, the presence of Co on the surface is generally preferred relative to Fe and the same for Fe relative to Cr. This observation is in line with trends in their surface energies (Cr > Fe > Co). However, for alloys with catalytically active elements, higher fractions of Ni and active elements facilitate more stable surfaces. This observation is consistent for all surface motifs. The surface segregation of Pd and Rh in Ni-based alloys has been reported previously^88–91^. In addition, surface energies of (100) and (211) facets are higher compared to (111) facets for all alloys in general, which corroborates the intuition that a higher coordination environment is more stable.

### Reactivity analysis of Ni-based alloys

The preceding analysis points to the favorable surface compositions for all considered alloys but does not inform on the distribution of possible sites on the surface, which would be plausible locations for catalytic activity. Here, we conduct this analysis by invoking identical assumptions as for the monometallic surfaces. Adsorbates are treated as occupying the fcc hollow, 4-fold hollow and a combination of 5-fold hollow sites on fcc(111), (100), and (211) surfaces. However, evaluating sites on alloy surfaces is nontrivial owing to the heterogeneity of site distributions. As a first approximation, we approach this by classifying sites solely based on identity of composing atoms. We do not distinguish between the coordination shells around the site atoms. For all surfaces, we also do not distinguish between the relative arrangement of site atoms. The number of possible unique fcc or 4-fold sites are 10 and 15 on the fcc(111) and (100) surfaces, respectively. For the fcc(211) surface, we treat the site ensemble as a connection of two step atoms and two terrace atoms beneath them (Supplementary Fig. S13), yielding 15 unique ensembles.

We first analyze the conditional probability of these sites occurring on the surface. For that, we define the probability of a surface configuration of an alloy, through a Boltzmann-type distribution:

10 $${P}_{{A}_{x}{B}_{y}{C}_{z}}^{{Boltz},\,{surf},i}\left(T\right)=\frac{{e}^{-{F}_{{A}_{x}{B}_{y}{C}_{z}}^{{surf},i}/({k}_{B}T)}}{{\sum }_{i}{e}^{-{F}_{{A}_{x}{B}_{y}{C}_{z}}^{{surf},i}/({k}_{B}T)}}$$

where the denominator is summed over all sampled configurations. We evaluate this over all prior sampled ~200 inversion symmetric configurations. We subsequently define the probability of a site ensemble ${P}_{{S}}$ as:

11 $${P}_{S}=\sum _{i}{P}_{{A}_{x}{B}_{y}{C}_{z}}^{{Boltz},\,{surf},i}(T){{Fraction}}_{i}^{S}$$

This is the Boltzmann weighted expectation value of a site ensemble, S, occurring on the surface. Figure 4c illustrates the site probabilities for a NiCrPt(111) surface. We find that clusters of two or three Cr atoms are least likely to occur on the surface, consistent with its preference for the bulk location. The NiCrPt ensemble has the highest probability of occurrence, followed by sites involving clusters of Pt atoms. Supplementary Fig. S14 reports the probability distributions for fcc(111) surfaces of all alloys. For alloys without any active elements, the surface is compositionally mixed. The most probable site ensembles are consistent with the segregation tendencies described in the previous section. However, upon the introduction of the active element, the distribution of sites depends on the alloying element. In general, for NiCo or NiCr, introduction of the active element still offers a diversity of sites, suppressing the occurrence of clusters of the stabilizing element. The effect is sharply seen for NiCoPd, where sites including Co do not appear on the surface. For NiFe, the addition of the active element skews the distribution of active sites towards ensembles containing the active element combined with Ni.

The site distributions of fcc(100) and fcc(211) surfaces are reported in Supplementary Figs. S15, S16. Due to the many unique combinations, only the five highest probable sites are reported. Interestingly, the change in facet also changes the most probable site clusters. For instance, the combination of NiCr with Rh for a fcc(100) facet leads to site clusters excluding Cr, which contrasts with its behavior for fcc(111) surface. The addition of Pt and Pd also leads to enhancement of site ensembles with their presence in the distribution. Six alloy surfaces including active elements expose sites that exclude the stabilizing element. Interestingly, NiCoPd and NiFePd appear to be the only alloy combinations which singularly expose clusters of NiPd sites across the three considered facets.

We next inspect the reactivity of these alloys by evaluating the descriptor binding energies (O/N) for each site ensemble. In addition, the C adsorption energies were also evaluated (relative to CO and O_2_) per ensemble as a qualitative proxy for potential coke susceptibility. Carbon binding energies have been previously used as indicators for coking for nickel and nickel-based catalysts^92–95^. We note that this descriptor only delineates the thermodynamic tendency for the surfaces to retain carbonaceous species, which often correlate with the carbon adsorption energy^36^, and does not directly inform on the microkinetics of coke formation and removal, which is beyond the scope of the study. The adsorption energies were averaged over 20 site configurations of every alloy surface. Figure 4d depicts the ensemble-specific distribution of adsorption energies plotted on the volcano map for (111) surfaces (Fig. 3a). The adsorption energies were evaluated for distinct coordination environments around the site ensemble. The standard deviations of these points are within 0.2 eV, highlighting the effect of the nearest environment of a site. However, to a first approximation, the ensemble composition is still a valid classifier with distinctly spaced points. There appears to be a linear scaling relationship between N and O adsorption energies, which places a limitation on the reactivity of exposed sites. This holds true for all alloys and all surfaces, see Supplementary Figs. S17–S19. The adsorption energies for fcc(100) and (211) surfaces were computed in a similar manner to identify for the most probable surface site ensembles^96–98^.

To characterize reaction kinetics of all alloys, we add the critical assumption that the surface-specific scaling and TSS relationships derived in Figs. 1 and 2 also hold true for the considered alloys. The catalytic activity of the alloy is, thus, estimated through a site-wise ensemble averaging of the rates of NO decompositions.

12 $${r}_{{Alloy}}=\sum _{i}^{{sites}}{P}_{{site},i}\ast r({E}_{{ads},N,i},{E}_{{ads},O,i})$$

**Fig. 5:**
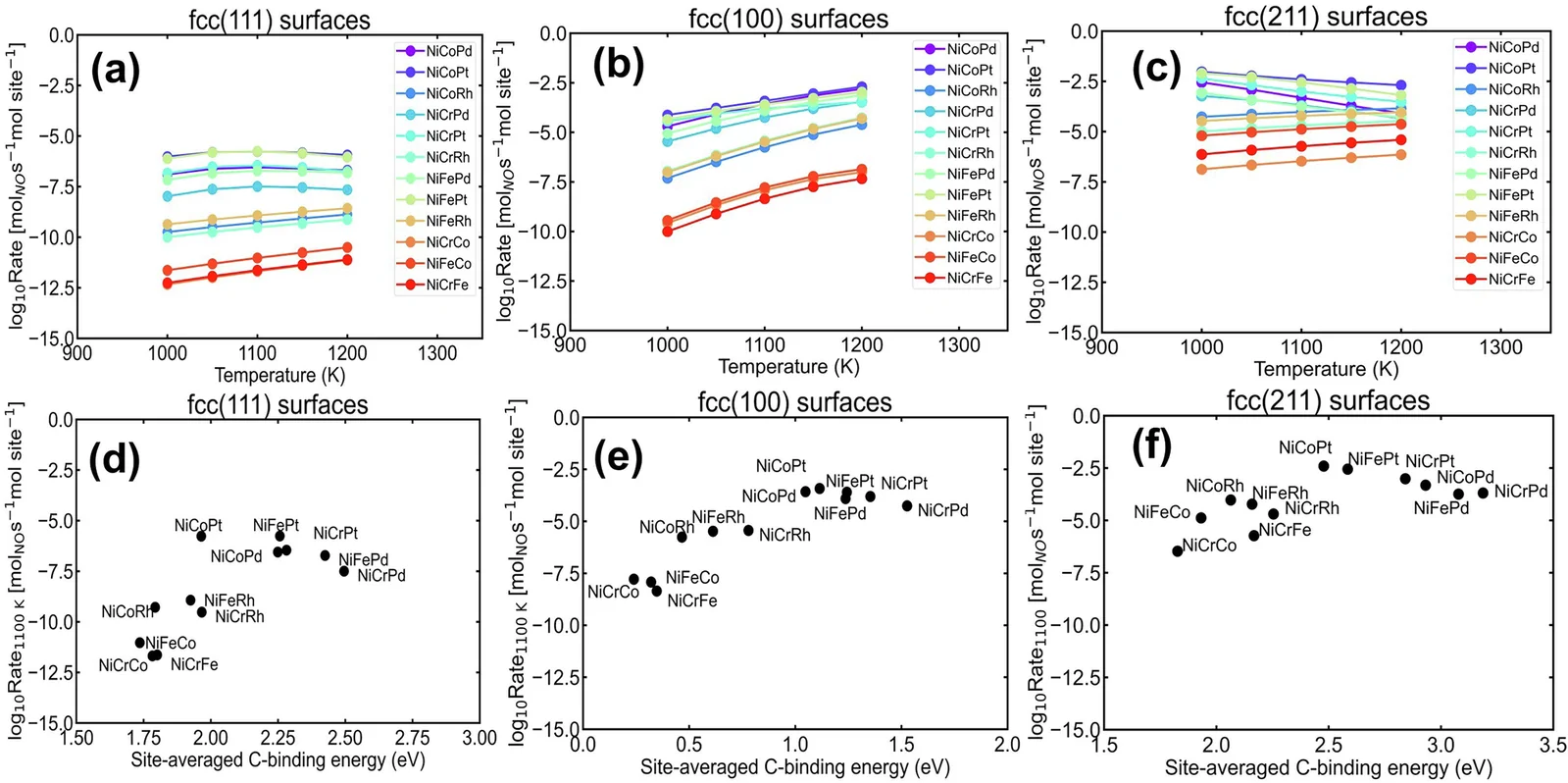
NO consumption rates on alloy fcc surfaces as a function of temperature and C binding energy. Panels (**a**–**c**) show the rate of NO consumption on the considered alloy fcc(111), fcc(100) and fcc(211) surfaces, respectively, as a function of temperature at *P*_N2_ = 0.7 bar, *P*_O2_ = 0.1 bar, *P*_CO_ = 0.0002 bar, *P*_NO_ = 0.002 bar, and *P*_CO2_ = 0.04 bar. Panels (**d**–**f**) show the rate of NO consumption on fcc(111) fcc(100) and fcc(211) surfaces, respectively, as a function of C-binding energy.

Figure 5a–c depicts the reaction rates of NO consumption on fcc(111), fcc(100), and fcc(211) alloy surfaces as a function of temperature at constant pressure conditions. The lowest rates consistently correspond to the alloys without active elements. The highest activity appears to favor the Pt-based alloys (closely followed by Pd) consistently with improvements up to six orders of magnitude in rates. Among all facets, the (211) structure exhibits the highest NO consumption rate for all alloys, followed by (100) and (111) motifs, underscoring the increased reactivity of corrugated surfaces exposing undercoordinated sites.

The C-binding energy was chosen as a descriptor to measure respective coking tendencies. The site-averaged C-binding energy of the alloy can be written as:

13 $${{BE}}_{C,{Alloy}}=\mathop{\sum}\limits_{i}^{{sites}}{P}_{{site},i}* {{BE}}_{C,i}$$

Figure 5d–f depicts the NO consumption rates as a function of the site-averaged C-binding energy. A nearly linear relationship is observed between both characteristics. This, thus, shows that the most active catalysts identified in our assessment, e.g., NiCrPt and NiCoPt, are also among those predicted to be less susceptible to deactivation due to surface coking within the present descriptor framework. This proportional relationship suggests that favorable NOx reduction activity and weaker carbon binding may be simultaneously accessible in this materials space, which could be useful for future catalyst design. However, we also note that the apparent absence of a trade-off in the present descriptor space should not be interpreted as proof that such a trade-off is absent under realistic operating conditions.

Here, we note again that microkinetic predictions have intrinsic uncertainties, as discussed in Section *Microkinetic model*, resulting from fitting in adsorbate scaling and transition-state scaling relationships. However, our aim is to identify clusters of compositions that consistently exhibit favourable activity within the descriptor framework. While uncertainties in the underlying energetics may shift the precise ordering of closely ranked systems, the overall structure of the activity landscape and the identification of promising compositional regions are likely to be more robust. The discovery of such promising material spaces would subsequently warrant high-fidelity calculations and further experimental validation.

We briefly comment here on the validity of the adsorption energies obtained using a non-spin-polarized treatment of the alloy surfaces. While this approximation is intended as a first-order representation of the high-temperature limit, where long-range magnetic ordering is not expected to persist, it does not explicitly capture local magnetic moments or magnetic disorder. To assess the sensitivity of the results to spin-polarization, we performed additional spin-polarized calculations for a representative (111) alloy surface while constraining the total magnetic moment to zero. For this surface, we recomputed the N and O adsorption energies for all site ensembles. Supplementary Figs. S21, S22 compare the adsorption energies obtained with and without spin-polarization. The largest deviations are observed for ensembles composed of magnetic elements, such as CoCoCo, FeFeFe, and CrCrCr, whereas ensembles involving nominally non-magnetic metals, such as Pd, Pt, and Rh, remain close to parity. Nevertheless, the overall correlations are largely preserved across all 12 alloy systems, with root-mean-square deviations comparable to typical DFT uncertainties of 0.1–0.2 eV. Supplementary Fig. S23 further shows that the site ensembles closest to the volcano maximum, and therefore associated with the highest predicted rates, exhibit only small deviations between the two treatments. These results suggest that the non-spin-polarized calculations are suitable for identifying broad screening trends, although quantitative analysis of magnetic alloys may require explicit spin-polarization. The present screening results should therefore be interpreted with this approximation in mind. More rigorous treatments of high-temperature magnetic disorder, such as DLM approaches^99,100^, could be applied in future work to refine the description of the most promising candidates.

## Discussion

NO_x_ emissions from aircraft exhausts have a range of deleterious effects on human health and air quality. Catalytic converters located at the exhaust provide a direct route for mitigating NO_x_ release. However, the design challenge for aircraft converters is substantial, owing to the extreme conditions of the exhaust and high exhaust velocities. This necessitates the exploration and design of active and stable materials, permitting the deployment of these technologies on a larger scale.

In this study, we conduct an in-silico design of potential catalytic candidates under exhaust conditions. We consider a reaction mechanism comprising concerted NO_x_ reduction and CO oxidation. We consider a range of transition metals and characterize the material space through the application of two descriptors, namely the O and N adsorption energies. We derive scaling relationships and TSS relationships to characterize the thermodynamic and kinetic quantities of this reaction as a function of the descriptors. Through mean-field microkinetic models, we establish structure-activity relationships in form of volcano maps, which provide a preliminary estimation of catalytic performance. We observe Pt, Pd, and Rh as the most active elements located near the volcano maximum in agreement with expectations.

We next considered Ni-based alloys and first evaluated the thermal stability of alloy materials including or excluding these active elements. We find that Cr is a potent stabilizing additive and its combination with Pt yields the most thermally stable bulk alloy. Studies of surface energies follow the same trends observed for bulk stability, with NiCrPt surfaces being consistently most stable across all considered crystallographic motifs. An analysis of reaction sites indicates that the most prevalent site clusters on the surface are a function of the facet and the alloying elements. However, the suppression of clusters involving the stabilizing metal upon introducing the active metal is consistently observed for all cases. Following reactivity analysis, we show that the introduction of active metals confers up to six orders of magnitude improvement in the catalytic activity and a concomitant enhanced predicted resistance to carbon retention. The Pt-based alloys are among the most active, closely followed by the Pd-based alloys. We finally propose NiCrPt as the lead candidate, demonstrating exceptional stability and catalytic function, closely followed by NiCoPd, NiCoPt, and NiFePt. In addition, this alloy is the least expensive material among the considered alloys containing active metals, making it more economically viable for large-scale applications. We strongly encourage future macroscale simulations or experimental studies evaluating the benefit of applying this catalyst as a surface coating on engine turbine parts (e.g., the stator and rotor) for the reduction of NO_x_ emissions from aircraft.

This study demonstrates how careful consideration of thermal stability and catalytic activity is necessary, since both characteristics do not always proportionately correspond^40^. At the same time, the present framework is intended as a first-order high-throughput screening approach. The microkinetic model represents a deliberate simplification of the full chemical complexity of aircraft exhaust, the alloy structures are approximated as chemically disordered solid solutions, and carbon binding is treated as a first-order thermodynamic proxy for the tendency to retain carbonaceous species. These approximations make it possible to traverse a broad alloy space, but they also imply that the present results should be interpreted as identifying promising materials classes and compositional regions rather than definitive catalyst rankings. Future work should therefore refine the treatment of multicomponent exhaust chemistry, segregation and microstructural evolution, and coke formation and removal under realistic conditions. In this direction, the application of pre-trained machine learned potentials (MLP)^101^ could play a particularly promising role in accelerating computational studies of prospective materials.

Overall, this work provides a computationally tractable first step toward the design of durable catalysts for NO_x_ mitigation in jet-engine exhaust and helps pave the way for more realistic multiscale modeling and experimental evaluation of catalytic emission-control strategies for aviation.

## Methods

### Geometric relaxation details

Density functional theory (DFT) calculations were performed within a plane wave, supercell approach on the Vienna Ab-initio Simulation Package (VASP)^102,103^, using the projector augmented wave (PAW)^104,105^ method with the capabilities of Atomic Simulation Environment (ASE)^106^. The revised Perdew-Burke-Enzerhoff (RPBE) functional within the Generalized Gradient Approximation (GGA)^107^ was chosen to describe exchange and correlation, owing to its ability to reasonably describe the energetics of bonding at the surfaces and improved agreement with experimental chemisorption energies.

The following transition metal elements were considered in our work: Ag, Au, Co, Cu, Ir, Ni, Os, Pd, Pt, Re, Rh, and Ru. Assuming a face centered cubic (fcc) bulk structure for each element, the bulk lattice constants of all elements were computed with an energy cutoff of 520 eV and an (8×8×8) Monkhorst-Pack **k**-point grid^108^. For elements naturally exhibiting the fcc phase, the relaxed lattice constants overpredict the experimental lattice constants (Supplementary Fig. S1) within 10%, a common characteristic of GGA functionals. Subsequently, *p*(3×3) fcc(111), fcc(100), and fcc(211) slabs were constructed for each element. The (111) and (100) surfaces were modeled with four layers and the bottom two layers were frozen during all geometry optimizations to mimic the bulk. The (211) surface was modeled using four (111)-layers with the bottom two layers frozen during all geometry relaxations. A vacuum layer separation of ~15 Å was maintained between periodic images. In addition, dipole corrections were imposed to eliminate spurious electrostatic interactions between periodic images. A Gaussian smearing of 0.1 eV width was implemented. Geometry optimizations for all models are performed with an energy cutoff of 500 eV, a force convergence criterion of 0.01 eV Å^−1^ and a (6×6×1) **k**-point grid. Adsorption energies of C, N, O, CO, and NO were referenced to gas-phase CO, NO, O_2_ and N_2_. Gas phase species energies and vibrational spectra were evaluated using 21×22×23 Å cells with a 1×1×1 **k**-point grid. Spin polarization was implemented for O_2_ and NO to simulate open shell states. For bulk and surface optimizations of Ni-based alloys, we consider equal compositions and set the bulk lattice constant as the average of the lattice constants of the individual elements, following Vegard’s law^80^. While bulk optimizations were spin-polarized, the surface optimizations were non-spin-polarized. This choice was motivated by the focus of the present study on high-temperature catalytic conditions (~1100 K) relevant to aircraft exhaust, where transition-metal alloys are expected to exist in a paramagnetic state with negligible long-range magnetic ordering. Under such conditions, the net magnetic moment approaches zero due to thermal disorder. A non-spin-polarized treatment provides a first-order and feasible approximation to the high-temperature paramagnetic limit as has been used in previous studies^109^. The consequences of this approximation are further discussed in the results.

Alloy surface slab models were constructed as four-layer 3×3 supercells with inversion symmetry imposed to eliminate artificial dipole moments perpendicular to the surface. Atomic species were distributed across lattice sites to reproduce the target overall alloy composition while preserving inversion symmetry between the top and bottom surfaces. Although the total slab composition matches the corresponding bulk alloy composition, the elemental distribution was not constrained to be identical across individual layers. This approach enables sampling of diverse local surface environments while maintaining a consistent global composition. To assess the effect of allowing spin degrees of freedom, additional spin-polarized calculations were carried out with the total magnetic moment constrained to zero, corresponding to NUPDOWN = 0 in VASP. This setup was used as an approximate treatment of paramagnetic behavior. The results are shown in Supplementary Figs. S21–S23 and discussed in the Results section.

All optimized structures from this work are uploaded to the Cathub database (https://www.catalysis-hub.org/publications/GoswamiHigh-throughput2026)^110^.

### Transition state calculations

Transition state energies were calculated using the machine-learning enhanced nudged elastic band (ML-NEB) method introduced by Garrido Torres et al.^111^. The process of training and testing is done iteratively: images are evaluated, and NEBs are optimized on the predicted model in a single-image evaluation fashion. The process continues until the uncertainty on each image is below a convergence criterion of 0.05 eV. Once this criterion is met, the predicted saddle point image is converged using the improved dimer method by Heyden et al.^112^, as implemented in VASP, to forces below 0.05 eV/Å. We computed vibrational frequencies for each transition state to verify the presence of a single imaginary mode.

### Microkinetic model

We focus on NO reduction with CO as reductant in this work:

14 $${NO}\left(g\right)+{CO}\left(g\right)\to \frac{1}{2}{N}_{2}\left(g\right)+{{CO}}_{2}\left(g\right)$$

For the above reaction, we consider the following reaction mechanism:

15 $${NO}\left(g\right)+* \leftrightarrow {{NO}}^{* }$$

16 $${CO}\left(g\right)+* \leftrightarrow {{CO}}^{* }$$

17 $$N{O}^{* }+* \leftrightarrow {N}^{* }+{O}^{* }$$

18 $$C{O}^{* }+* \leftrightarrow {C}^{* }+{O}^{* }$$

19 $$C{O}^{* }+{O}^{* }\leftrightarrow {{CO}}_{2}\left(g\right)+{2}^{* }$$

20 $${O}^{* }+{O}^{* }\leftrightarrow {O}_{2}\left(g\right)+{2}^{* }$$

21 $${N}^{* }+{N}^{* }\leftrightarrow {N}_{2}\left(g\right)+{2}^{* }$$

This mechanism integrates CO oxidation and NO reduction. We presently omit hydrocarbon oxidation, which can play a significant role at higher temperatures^113^ since our focus is on the core function of the catalyst. A self-consistent mean-field microkinetic model^114,115^ was generated and numerically solved through CatMap^116^, which constructs coupled differential equations and solves using the steady-state approximation. The entropy of gas-phase species was approximated by the Shomate equation with parameters taken from the NIST-JANAF Thermochemical Tables^117^. The entropy and zero-point energy of adsorbed species was taken for Pt surfaces only and evaluated within the harmonic approximation using central differences with a magnitude of displacements of 0.01 Å. During vibrational analysis of adsorbates, the surfaces were fixed. The entropy estimates were used to parameterize the pre-factors of the rate equations.

## Supplementary information

NOx_EmissionControl_SI_npj_upd

## Data Availability

All results reported in this article are based on optimized structures and corresponding energies deposited in the CatHub database at https://www.catalysis-hub.org/publications/GoswamiHigh-throughput2026.
