# Supplementary material for: High-throughput design of catalytic materials for NOx reduction from aircraft emissions
Source: NPJ Comput Mater. 2026 Jul 9;12(1):363. doi: 10.1038/s41524-026-02220-9 (PMC13727672; doi:10.1038/s41524-026-02220-9)
Supplement: Supplementary file 1 [file 41524_2026_2220_MOESM1_ESM.pdf]

**SUPPLEMENTARY INFORMATION FOR:**

# High-throughput design of catalytic materials for $NO_x$ reduction from aircraft emissions


Anshuman Goswami[1,2,3], Frank Abild-Pedersen [1,2*], John W. Lawson[4*], and Joakim Halldin Stenlid [3,5*]

[1]*Department of Chemical Engineering, Stanford University, 443 Via Ortega, Stanford, CA 94305, USA*

[2]*SUNCAT Center for Interface Science and Catalysis, SLAC National Accelerator Laboratory, 2575 Sand Hill Road, Menlo Park, CA 94025, USA*

[3]*KBR, Inc., Intelligent Systems Division, NASA Ames Research Center, Moffett Field, CA, 94035, USA*

[4]*Intelligent Systems Division, NASA Ames Research Center, Moffett Field, CA, 94035, USA*

[5]*Department of Chemistry and Chemical Engineering, Chalmers University of Technology, Kemivägen 10 ,Gothenburg, SE-412 96, Sweden*

[*] *Corresponding e-mail address: abild@slac.stanford.edu*

*Corresponding e-mail address: john.w.lawson@nasa.gov*

*Corresponding e-mail address: stenlid@chalmers.se*

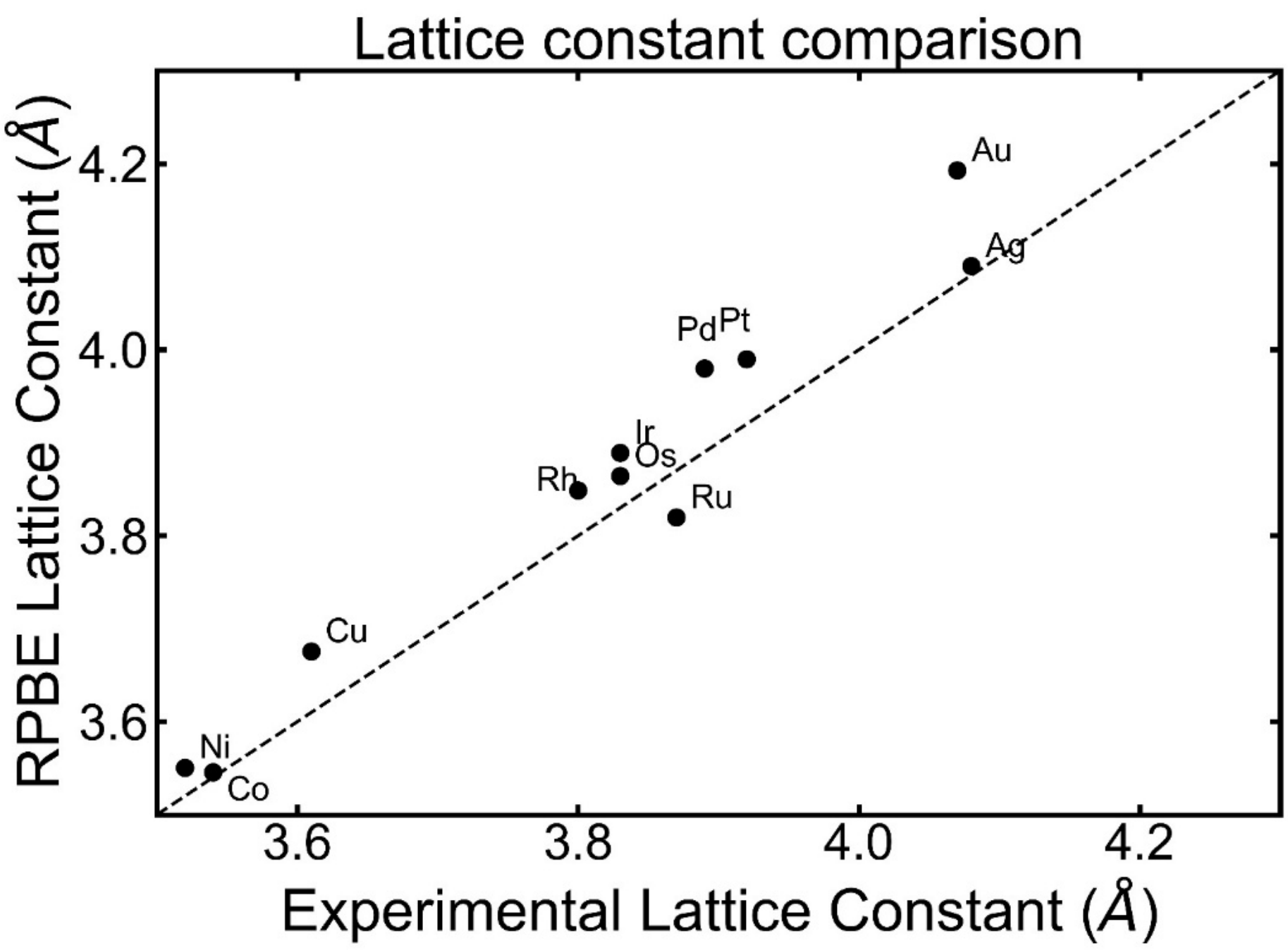


**Figure S1. Lattice constants from DFT vs Experiments**: Parity plot of lattice constants predicted using RPBE functional against experimental lattice constants for transition metals assuming face-centered cubic (fcc) structure.

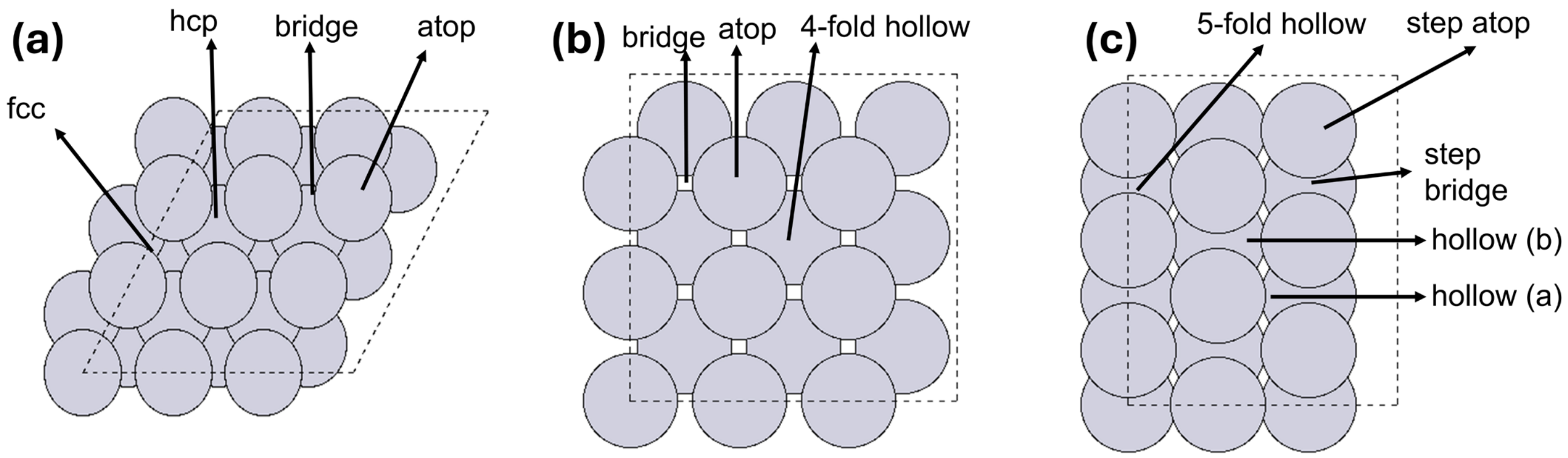


**Figure S2. Adsorption sites on crystallographic facets**: Schematic illustration of adsorption sites on (a) fcc(111) (b) fcc(100) and (c) fcc(211) crystallographic surfaces. In the figure, fcc is face-centered cubic, hcp means hexagonal closed-packed.

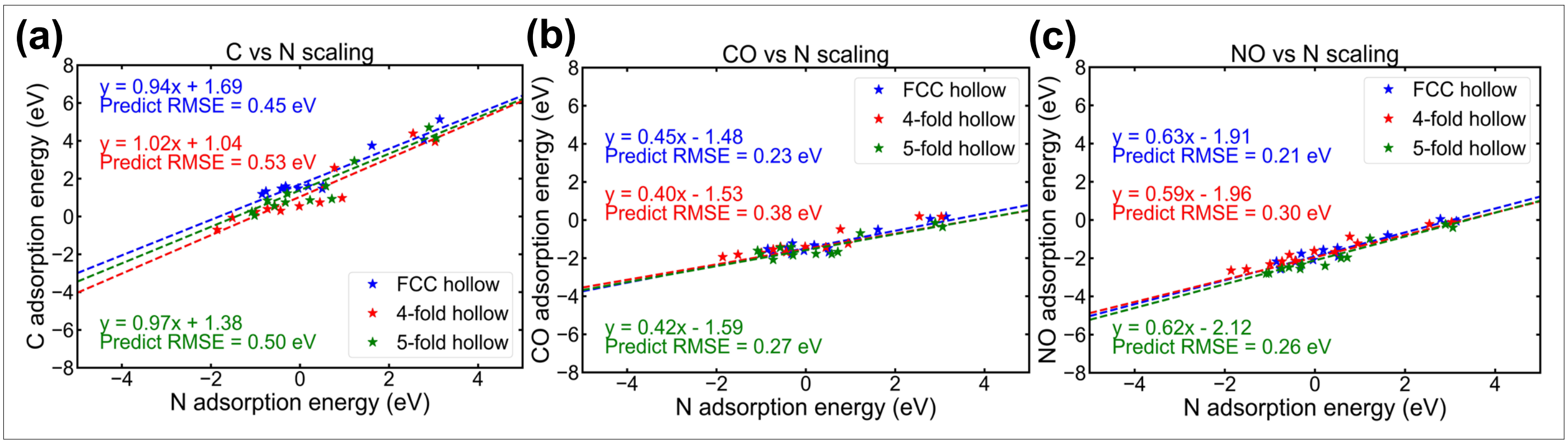


**Figure S3**. **Accuracy of linear scaling relationships:** Prediction agreement of scaling relationships against (a) C vs N, (b) CO vs N and (c) NO vs N adsorption energies on Ag/Au/Co/Cu/Ir/Ni/Os/Pd/Pt/Re/Rh/Ru surfaces. Blue, red and green data points correspond to adsorption energies computed on fcc hollow on fcc(111), 4-fold hollow from fcc(100) and 5-fold hollow sites from fcc(211) surface of considered metals. Dashed lines correspond to surface-wise linear relations from Figure 1 of the main text. Root mean squared errors of agreement (in eV) between the lines and data points are indicated. In the figure, fcc is face-centered cubic.

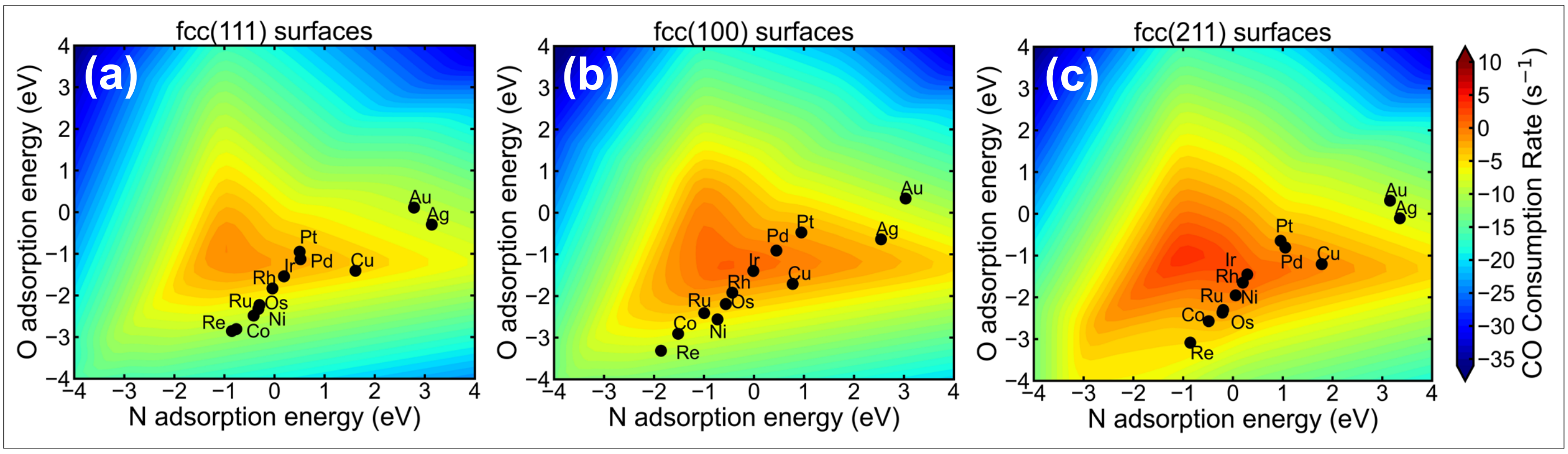


**Figure S4. CO decomposition activity contour maps**: CO decomposition rate as a function of O and N binding energies on (a) fcc(111) (b) fcc(100) and (c) fcc(211) surfaces, evaluated through a mean-field microkinetic model at T = 1100 K, $P_{N2}$ = 0.7 bar, $P_{O2}$ = 0.1 bar, $P_{CO}$ = 0.0002 bar, $P_{NO}$ = 0.002 bar and $P_{CO2}$ = 0.04 bar. Data points correspond to adsorption energies computed on fcc hollow/4-fold hollow/combination of step and five-fold hollow sites for fcc(111)/fcc(100)/fcc(211) surfaces of every metal. In the figure, fcc is face-centered cubic, T is temperature, and $P_x$ partial pressures.

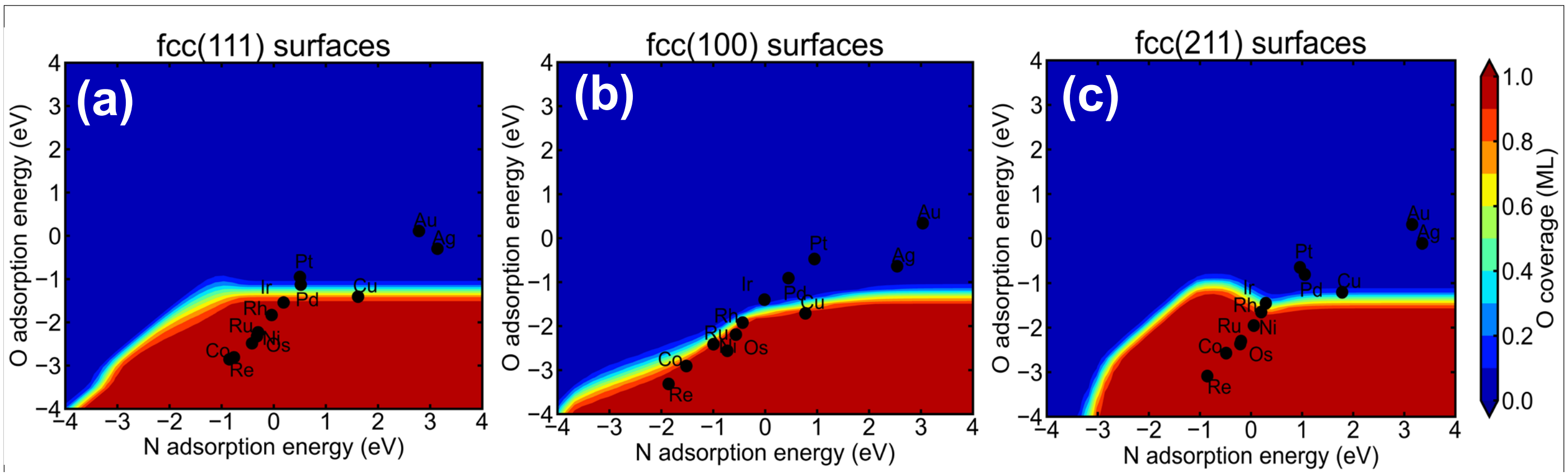


**Figure S5: Oxygen coverage contour maps**: O coverage as a function of O and N binding energies on (a) fcc(111) (b) fcc(100) and (c) fcc(211) surfaces, evaluated through a mean-field microkinetic model at T = 1100 K, $P_{N2}$ = 0.7 bar, $P_{O2}$ = 0.1 bar, $P_{CO}$ = 0.0002 bar, $P_{NO}$ = 0.002 bar and $P_{CO2}$ = 0.04 bar. Data points correspond to adsorption energies computed on fcc hollow/4-fold hollow/combination of step and five-fold hollow sites for fcc(111)/fcc(100)/fcc(211) surfaces of every metal. In the figure, fcc is face-centered cubic, T is temperature, and Px partial pressures. ML corresponds to a monolayer.

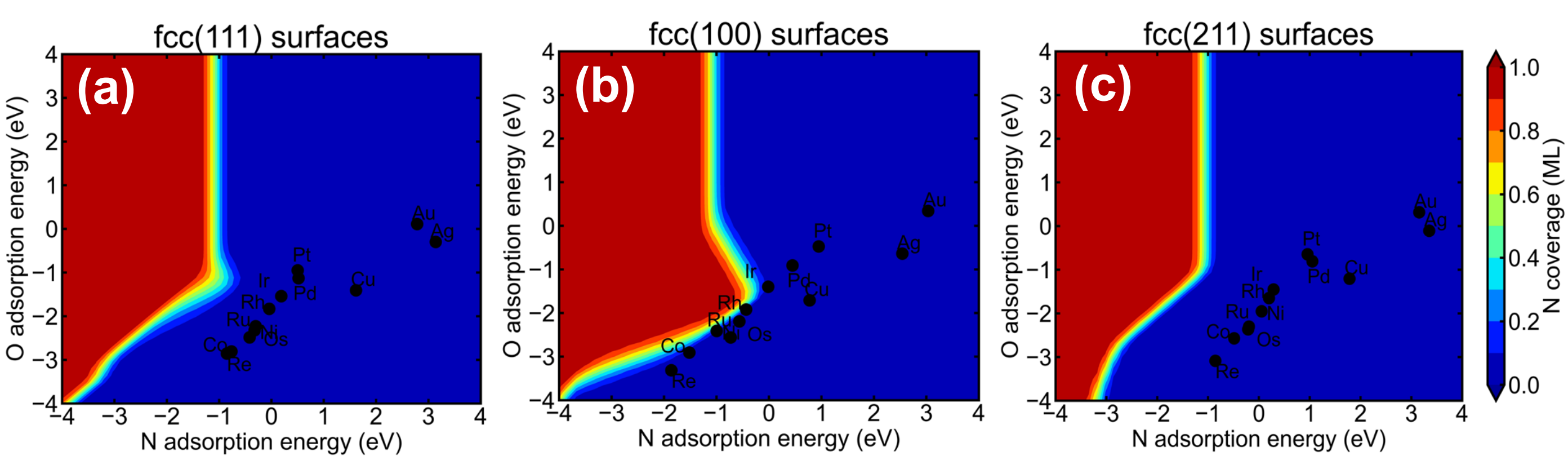


**Figure S6: Nitrogen coverage contour maps**: N coverage as a function of O and N binding energies on (a) fcc(111) (b) fcc(100) and (c) fcc(211) surfaces, evaluated through a mean-field microkinetic model at T = 1100 K, $P_{N2}$ = 0.7 bar, $P_{O2}$ = 0.1 bar, $P_{CO}$ = 0.0002 bar, $P_{NO}$ = 0.002 bar and $P_{CO2}$ = 0.04 bar. Data points correspond to adsorption energies computed on fcc hollow/4-fold hollow/combination of step and five-fold hollow sites for fcc(111)/fcc(100)/fcc(211) surfaces of every metal. In the figure, fcc is face-centered cubic, T is temperature, and Px partial pressures. ML corresponds to a monolayer.

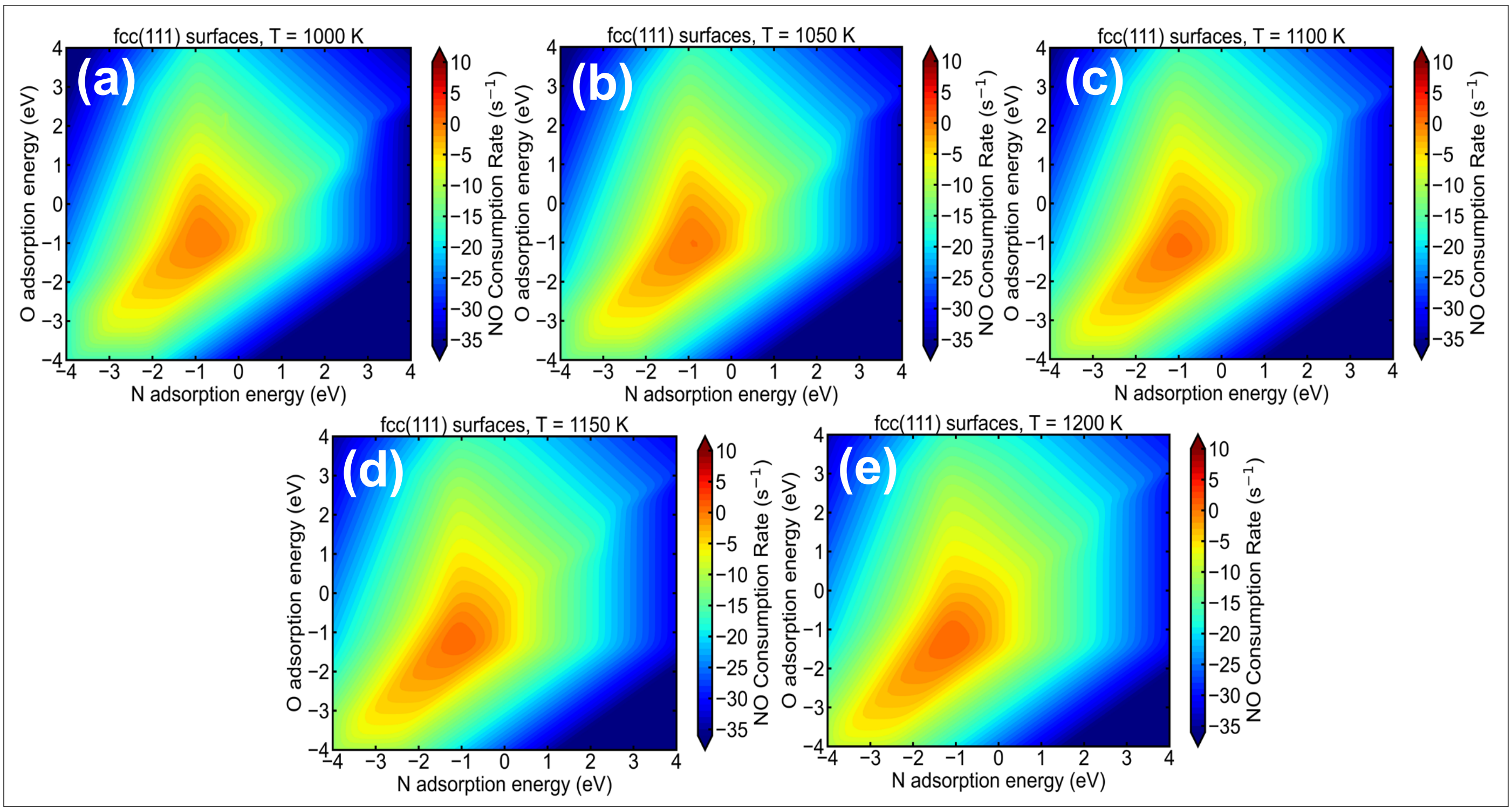


**Figure S7. Temperature dependent NO decomposition activity contour maps on fcc(111) surfaces:** (a-e) NO consumption rate as a function of N and O adsorption energies on fcc(111) surfaces at temperatures of 1000, 1050, 1100, 1150, and 1200 K, with $P_{N2}$ = 0.7 bar, $P_{O2}$ = 0.1 bar, $P_{CO}$ = 0.0002 bar, $P_{NO}$ = 0.002 bar and $P_{CO2}$ = 0.04 bar. In the figure, fcc is face-centered cubic, T is temperature, and Px partial pressures.

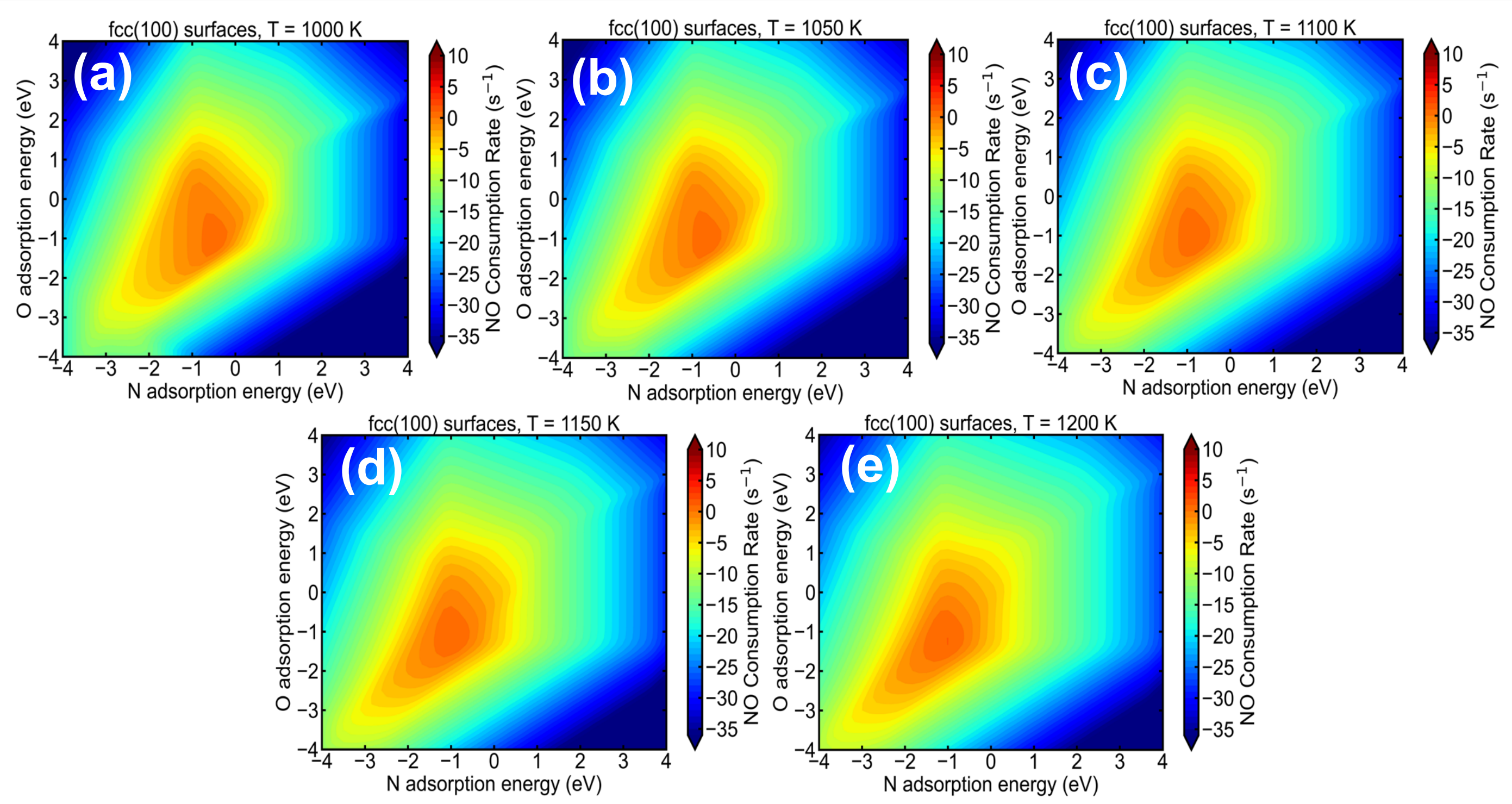


**Figure S8. Temperature dependent NO decomposition activity contour maps on fcc(100) surfaces**: (a-e) NO consumption rate as a function of N and O adsorption energies on fcc(100) surfaces at temperatures of 1000, 1050, 1100, 1150, and 1200 K, with $P_{N2}$ = 0.7 bar, $P_{O2}$ = 0.1 bar, $P_{CO}$ = 0.0002 bar, $P_{NO}$ = 0.002 bar and $P_{CO2}$ = 0.04 bar. In the figure, fcc is face-centered cubic, T is temperature, and Px partial pressures.

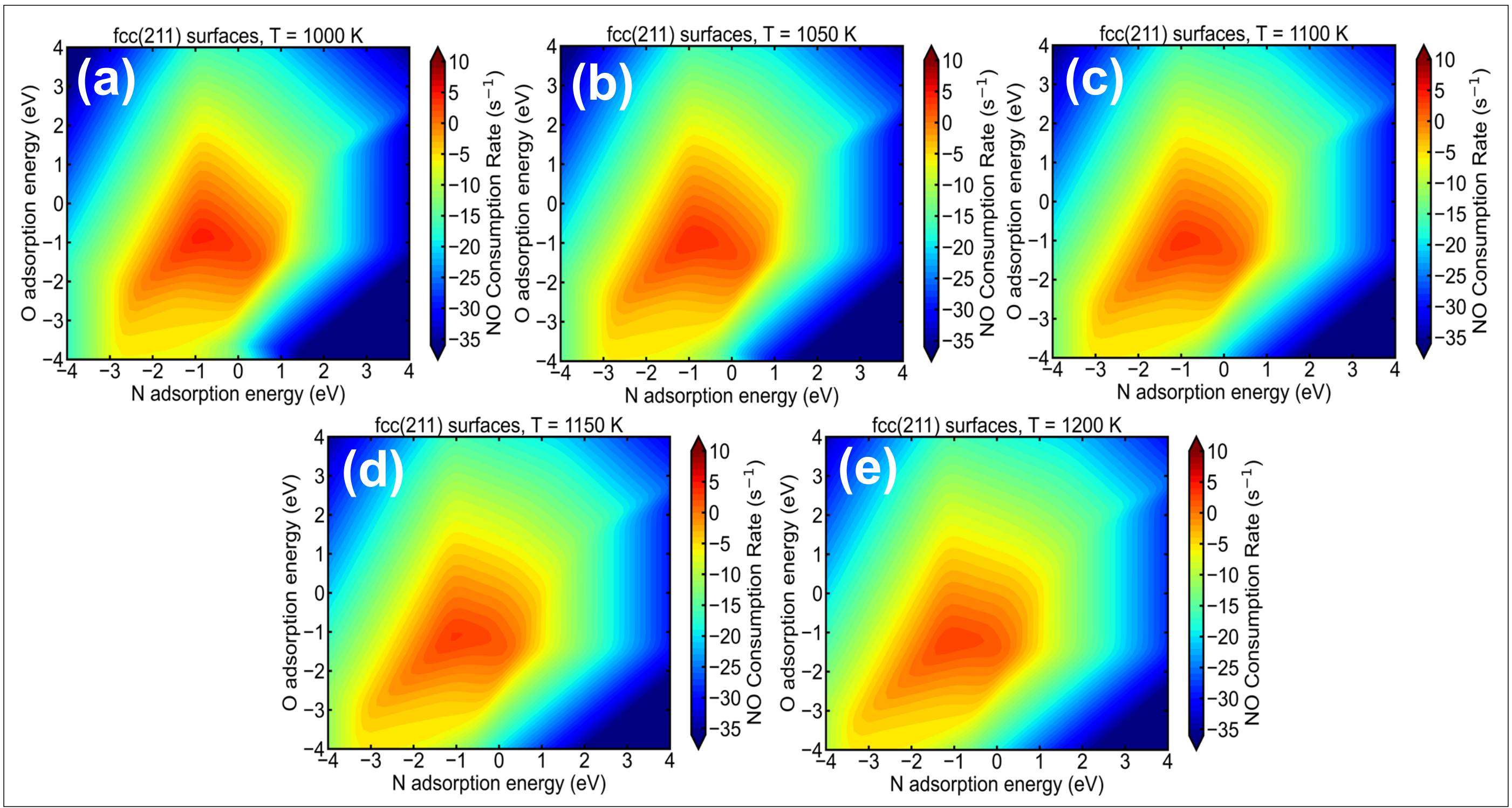


**Figure S9**. **Temperature dependent NO decomposition activity contour maps on fcc(211) surfaces:** (a-e) NO consumption rate as a function of N and O adsorption energies on fcc(211) surfaces at temperatures of 1000, 1050, 1100, 1150, and 1200 K, with $P_{N2}$ = 0.7 bar, $P_{O2}$ = 0.1 bar, $P_{CO}$ = 0.0002 bar, $P_{NO}$ = 0.002 bar and $P_{CO2}$ = 0.04 bar. In the figure, fcc is face-centered cubic, T is temperature, and $P_x$ partial pressures.

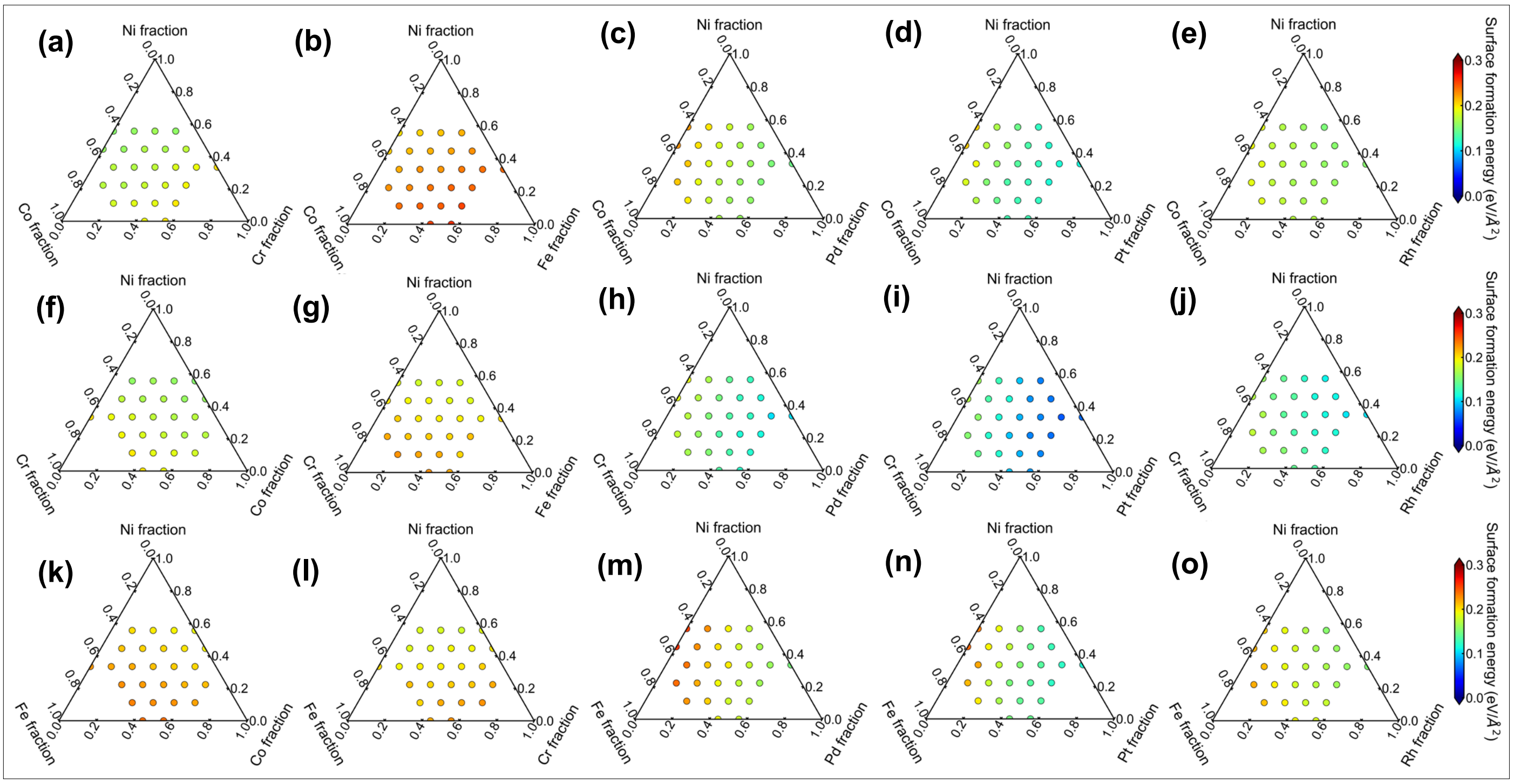


**Figure S10. Surface-composition wise formation energies of alloy fcc(111) surfaces**: Ternary diagrams depicting the formation energies of fcc(111) surfaces of (a) NiCoCr (b) NiCoFe (c) NiCoPd (d) NiCoPt (e) NiCoRh (f) NiCrCo (g) NiCrFe (h) NiCrPd (i) NiCrPt (j) NiCrRh (k) NiFeCo (l) NiCrFe (m) NiFePd (n) NiFePt and (o) NiFeRh, as a function of surface composition of constituent metals. In the figure, fcc is face-centered cubic.

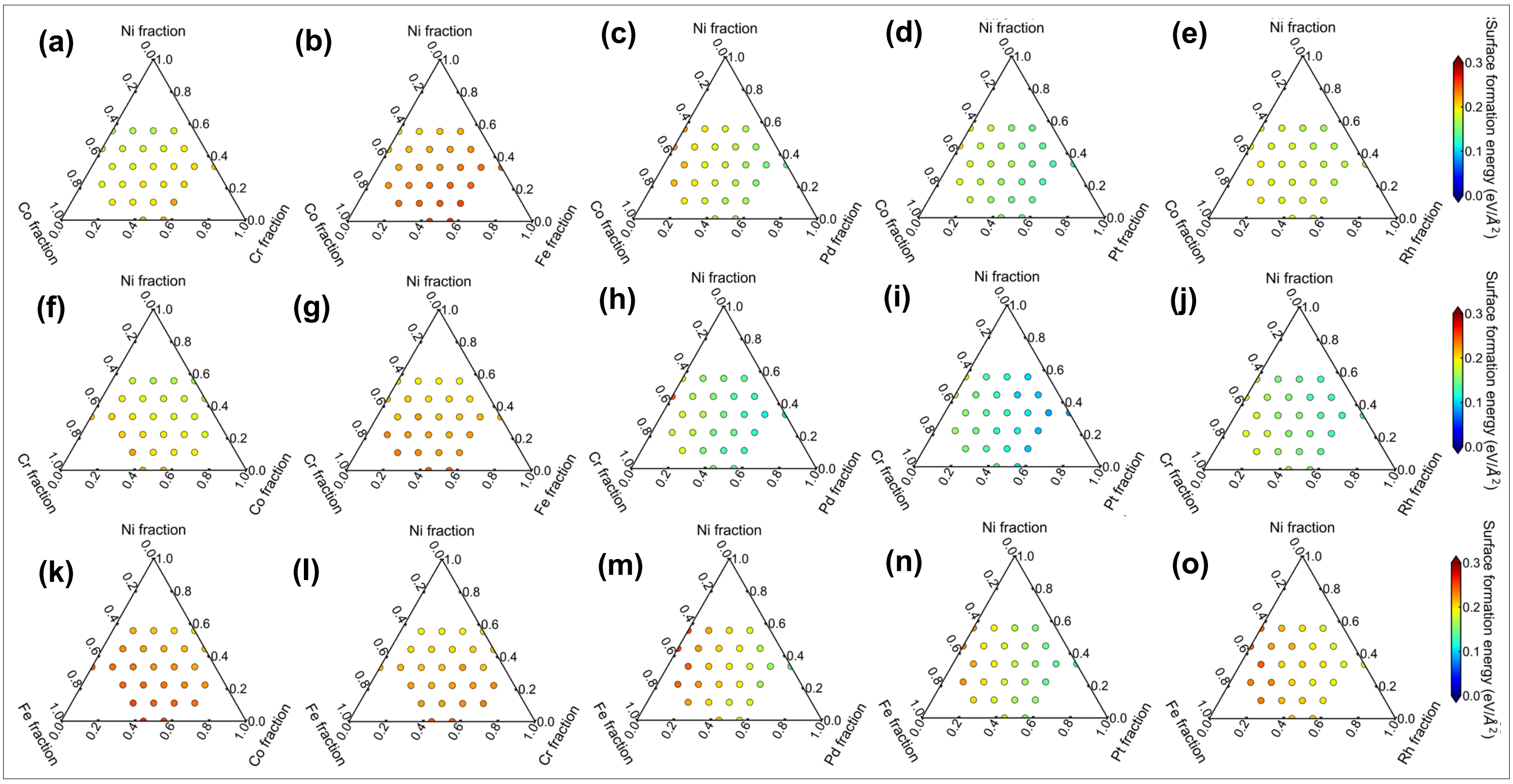


**Figure S11. Surface-composition wise formation energies of alloy fcc(100) surfaces**: Ternary diagrams depicting the formation energies of fcc(100) surfaces of (a) NiCoCr (b) NiCoFe (c) NiCoPd (d) NiCoPt (e) NiCoRh (f) NiCrCo (g) NiCrFe (h) NiCrPd (i) NiCrPt (j) NiCrRh (k) NiFeCo (l) NiCrFe (m) NiFePd (n) NiFePt and (o) NiFeRh, as a function of surface composition of constituent metals. In the figure, fcc is face-centered cubic.

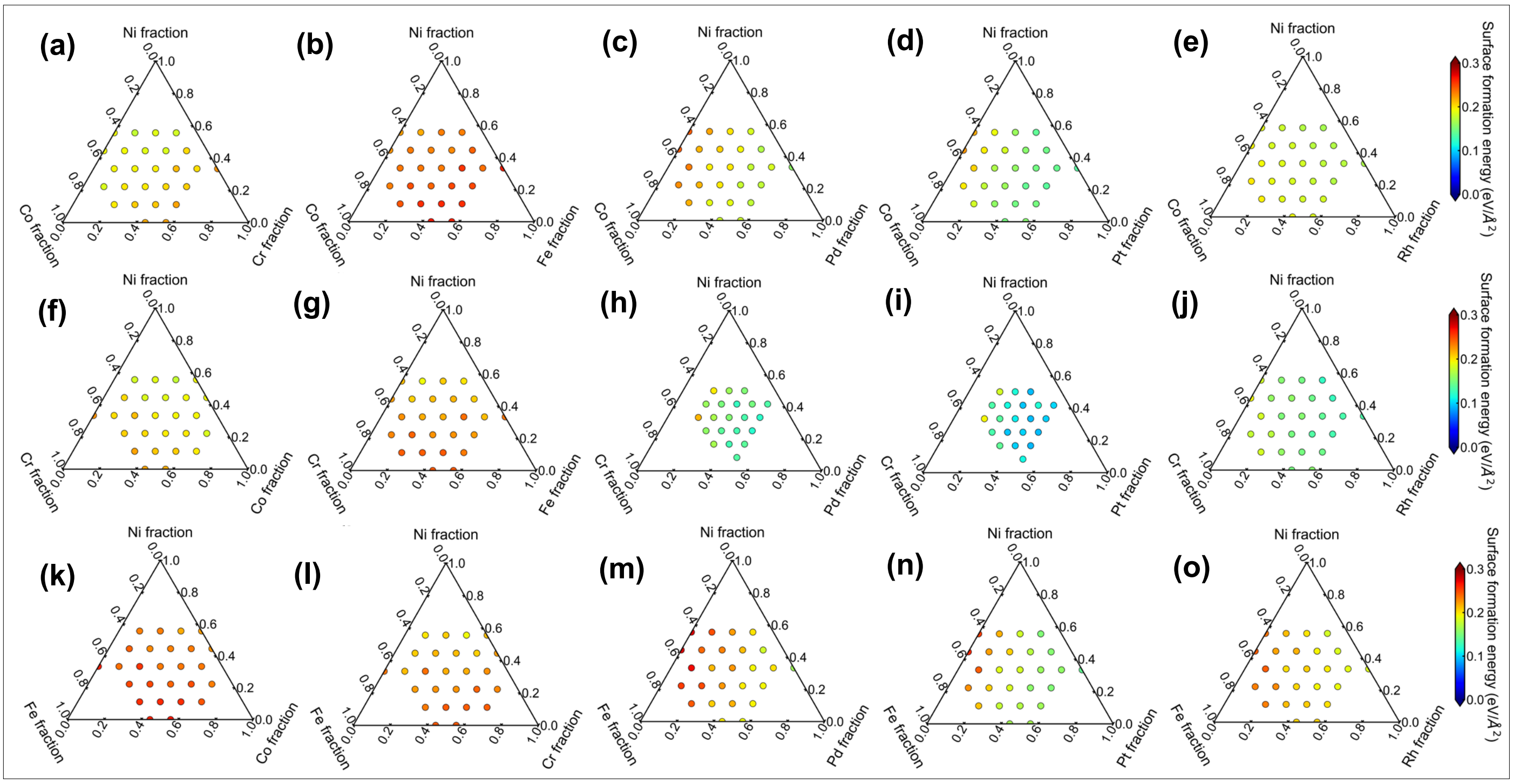


**Figure S12. Surface-composition wise formation energies of alloy fcc(211) surfaces**: Ternary diagrams depicting the formation energies of fcc(211) surfaces of (a) NiCoCr (b) NiCoFe (c) NiCoPd (d) NiCoPt (e) NiCoRh (f) NiCrCo (g) NiCrFe (h) NiCrPd (i) NiCrPt (j) NiCrRh (k) NiFeCo (l) NiCrFe (m) NiFePd (n) NiFePt and (o) NiFeRh, as a function of surface composition of constituent metals. In the figure, fcc is face-centered cubic.

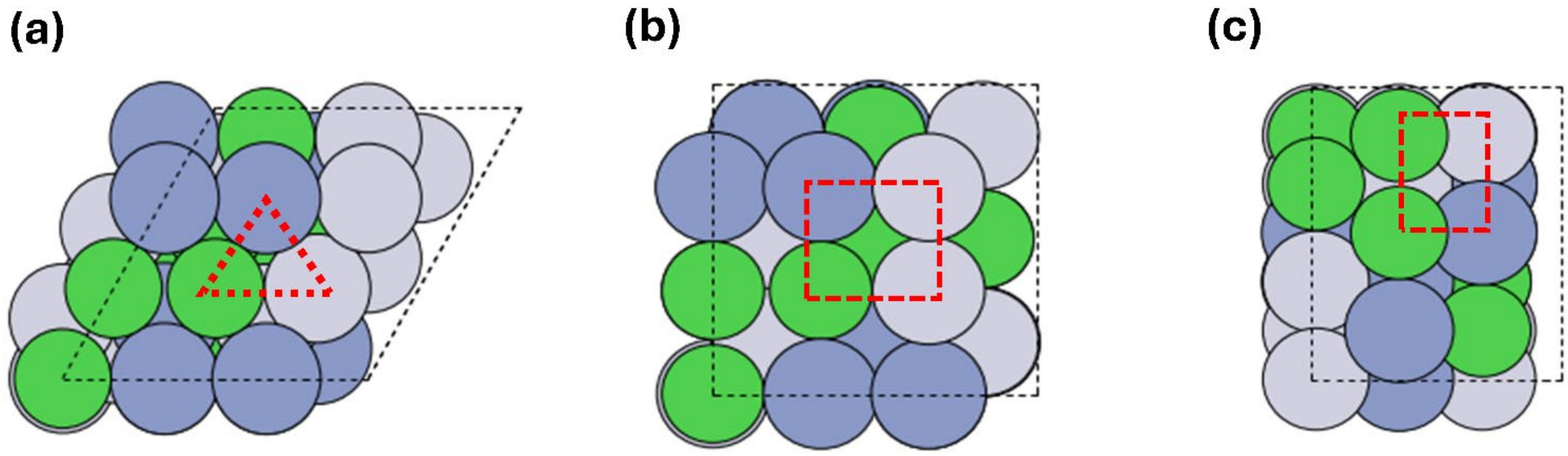


**Figure S13. Schematic representations of sites considered for alloy surfaces:** Surface representations of (a) fcc(111) (b) fcc(100) and fcc(211) surface of a NiCrPt alloy. Grey/blue/green atoms represent Pt/Cr/Ni respectively. Sites encircled by triangles denote the CrNiPt, CrNiPtPt and CrNiNiPt site ensembles respectively. In the figure, fcc is face-centered cubic.

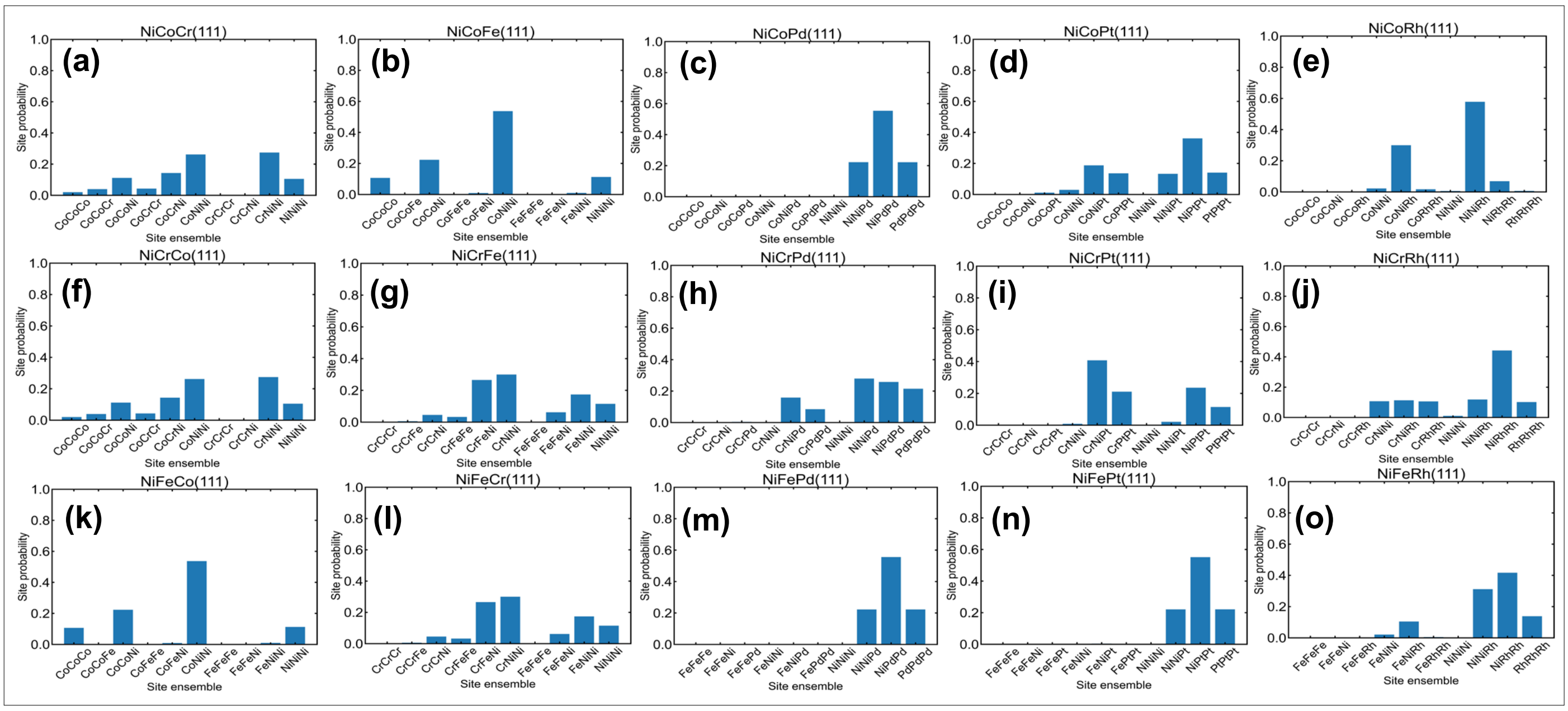


**Figure S14. Ensemble-wise probabilities of alloy fcc(111) surfaces**: Surface fraction of site ensembles on fcc(111) surfaces of (a) NiCoCr (b) NiCoFe (c) NiCoPd (d) NiCoPt (e) NiCoRh (f) NiCrCo (g) NiCrFe (h) NiCrPd (i) NiCrPt (j) NiCrRh (k) NiFeCo (l) NiCrFe (m) NiFePd (n) NiFePt and (o) NiFeRh. In the figure, fcc is face-centered cubic.

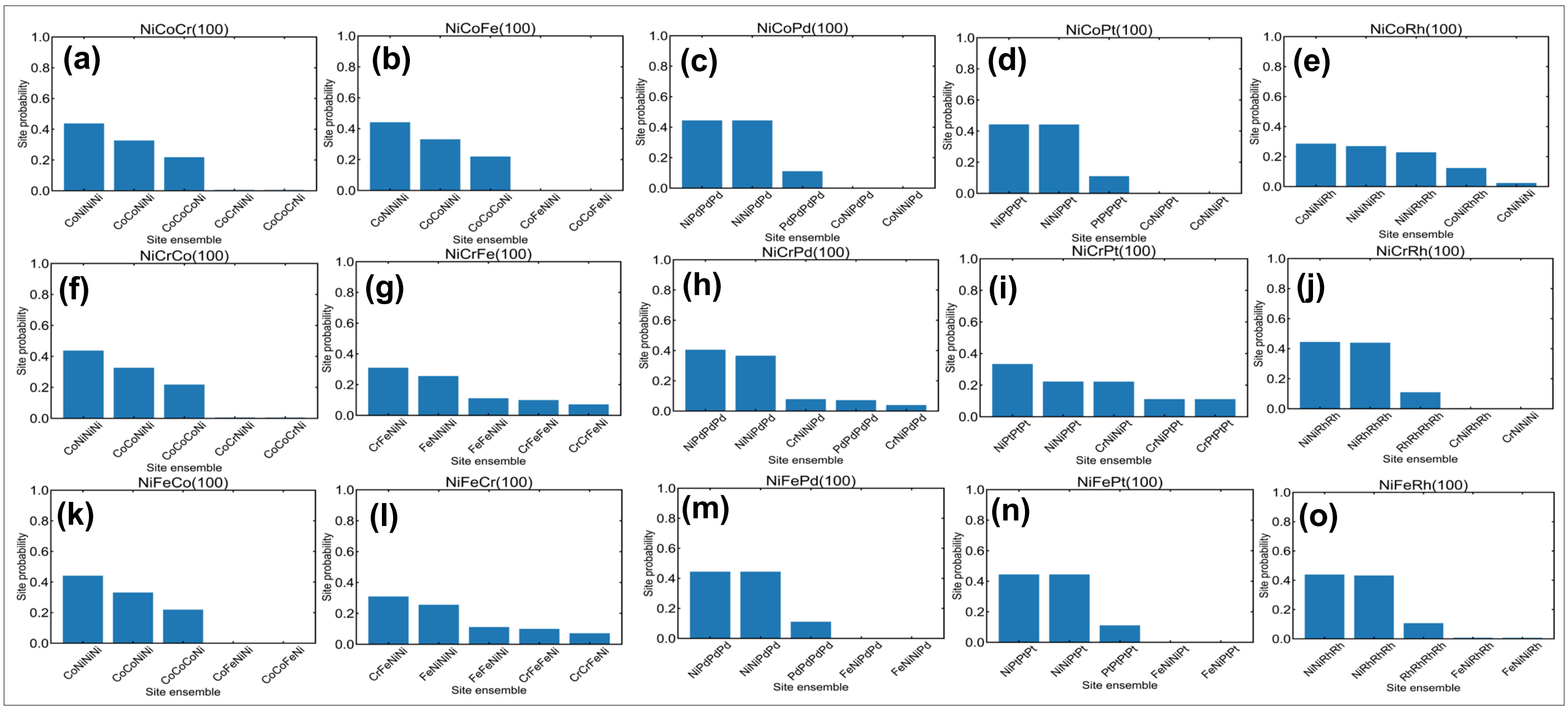


**Figure S15. Ensemble-wise probabilities of alloy fcc(100) surfaces**: Surface fraction of most probable site ensembles on fcc(100) surfaces of (a) NiCoCr (b) NiCoFe (c) NiCoPd (d) NiCoPt (e) NiCoRh (f) NiCrCo (g) NiCrFe (h) NiCrPd (i) NiCrPt (j) NiCrRh (k) NiFeCo (l) NiCrFe (m) NiFePd (n) NiFePt and (o) NiFeRh. In the figure, fcc is face-centered cubic.

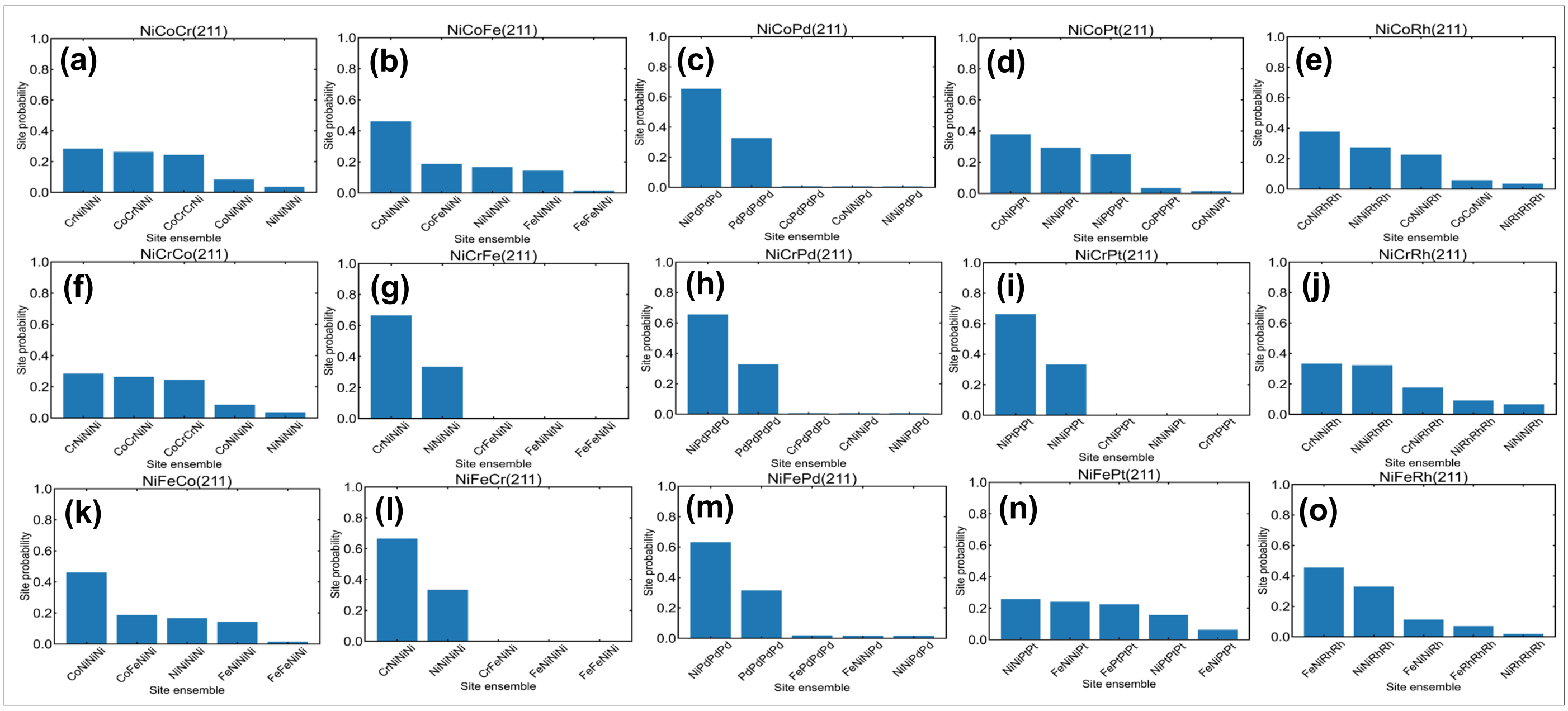


**Figure S16. Ensemble-wise probabilities of alloy fcc(211) surfaces**: Surface fraction of site ensembles of constituent metals on fcc(211) surfaces of (a) NiCoCr (b) NiCoFe (c) NiCoPd (d) NiCoPt (e) NiCoRh (f) NiCrCo (g) NiCrFe (h) NiCrPd (i) NiCrPt (j) NiCrRh (k) NiFeCo (l) NiCrFe (m) NiFePd (n) NiFePt and (o) NiFeRh. In the figure, fcc is face-centered cubic.

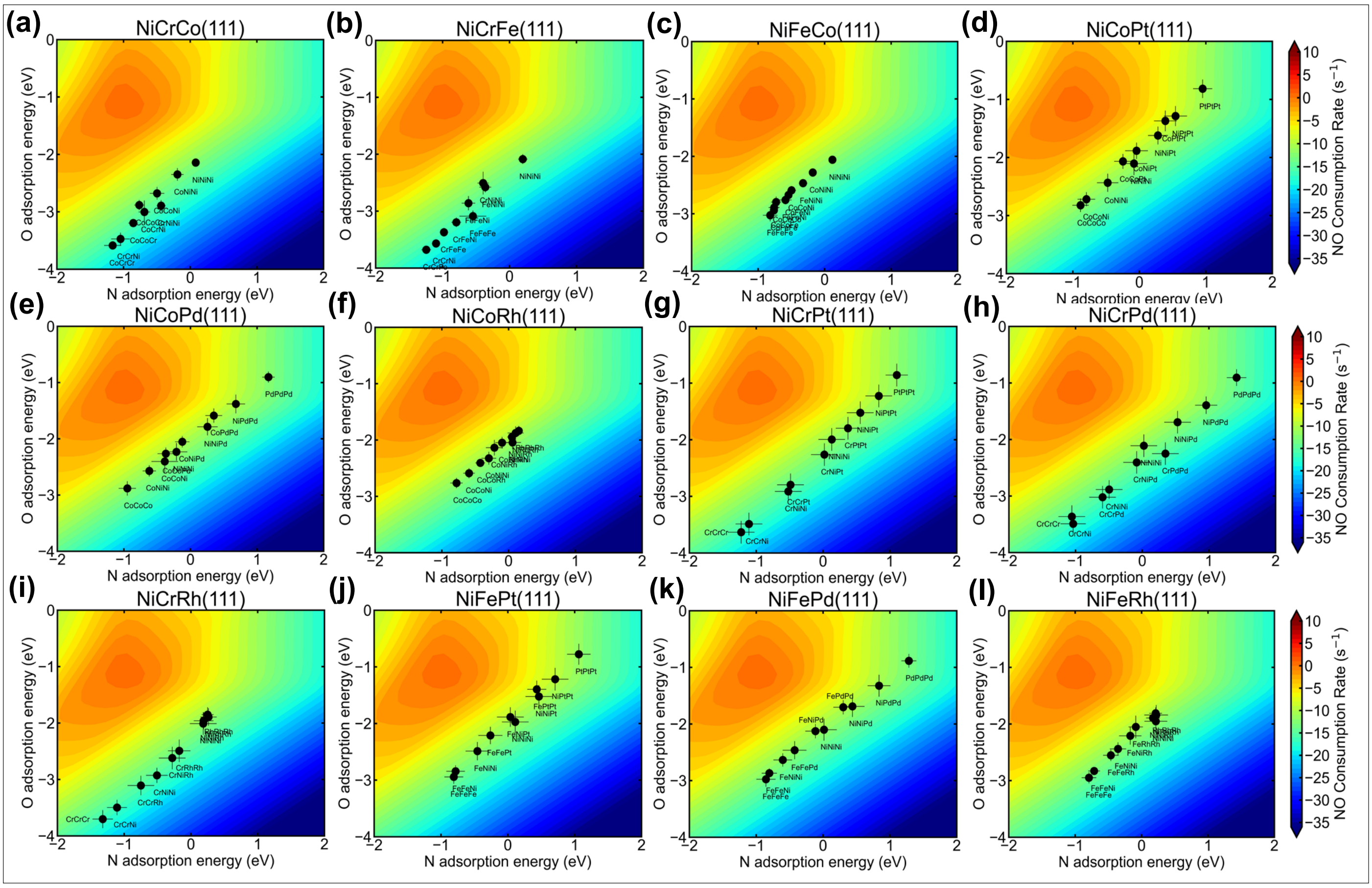


**Figure S17. Ensemble-wise adsorption energies and reaction rates for alloy fcc(111) surfaces:** Ensemble-wise adsorption energies overlaid on the NO consumption activity volcano for fcc(111) surfaces of (a) NiCrCo (b) NiCrFe (c) NiFeCo (d) NiCoPt (e) NiCoPd (f) NiCoRh (g) NiCrPt (h) NiCrPd (i) NiCrRh (j) NiFePt (k) NiFePd and (l) NiFeRh. Error bars indicate corresponding standard deviation. In the figure, fcc is face-centered cubic.

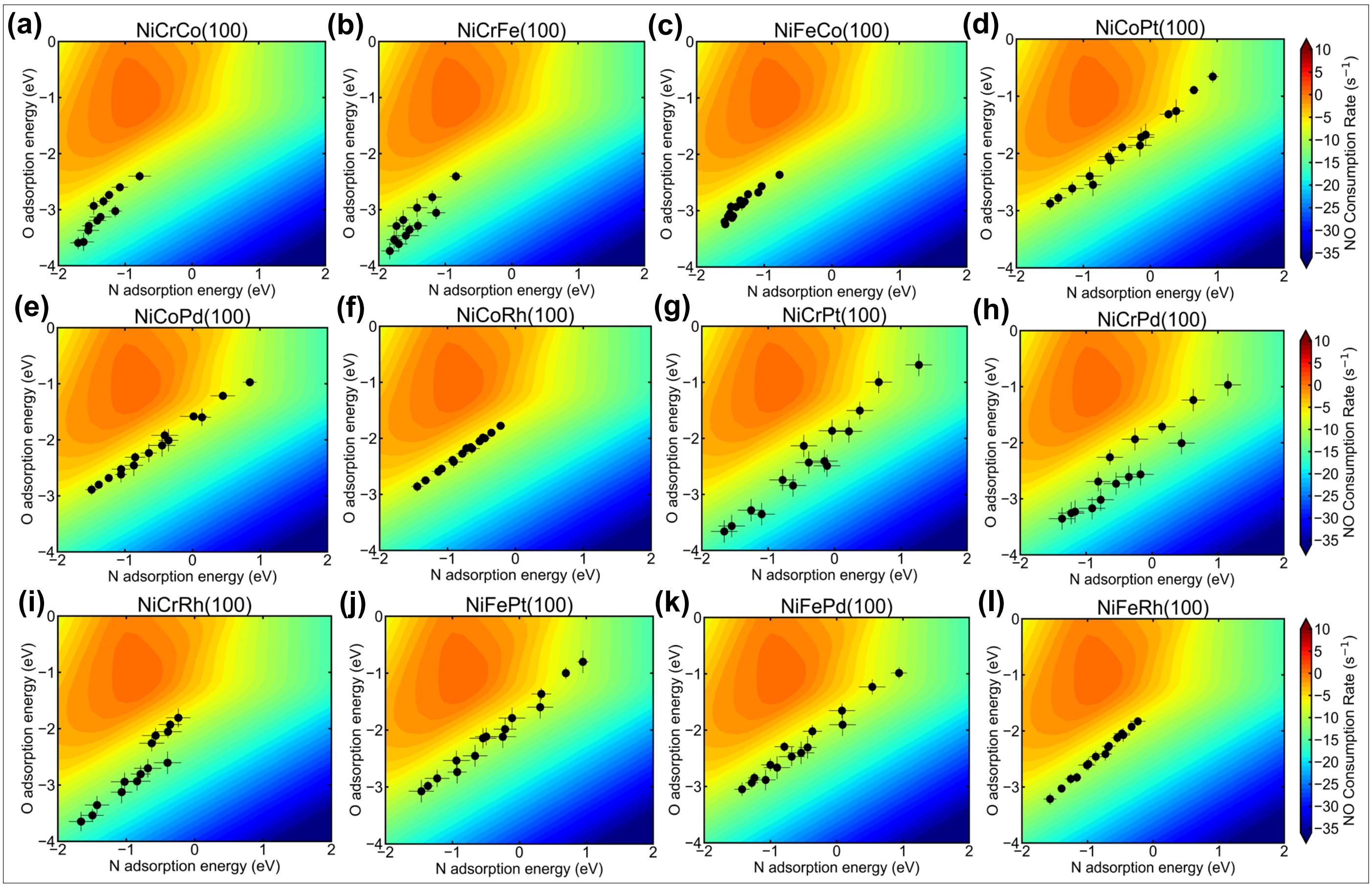


**Figure S18. Ensemble-wise adsorption energies and reaction rates for alloy fcc(100) surfaces:** Ensemble-wise adsorption energies overlaid on the NO consumption activity volcano for fcc(111) surfaces of (a) NiCrCo (b) NiCrFe (c) NiFeCo (d) NiCoPt (e) NiCoPd (f) NiCoRh (g) NiCrPt (h) NiCrPd (i) NiCrRh (j) NiFePt (k) NiFePd and (l) NiFeRh. Error bars indicate corresponding standard deviation. In the figure, fcc is face-centered cubic.

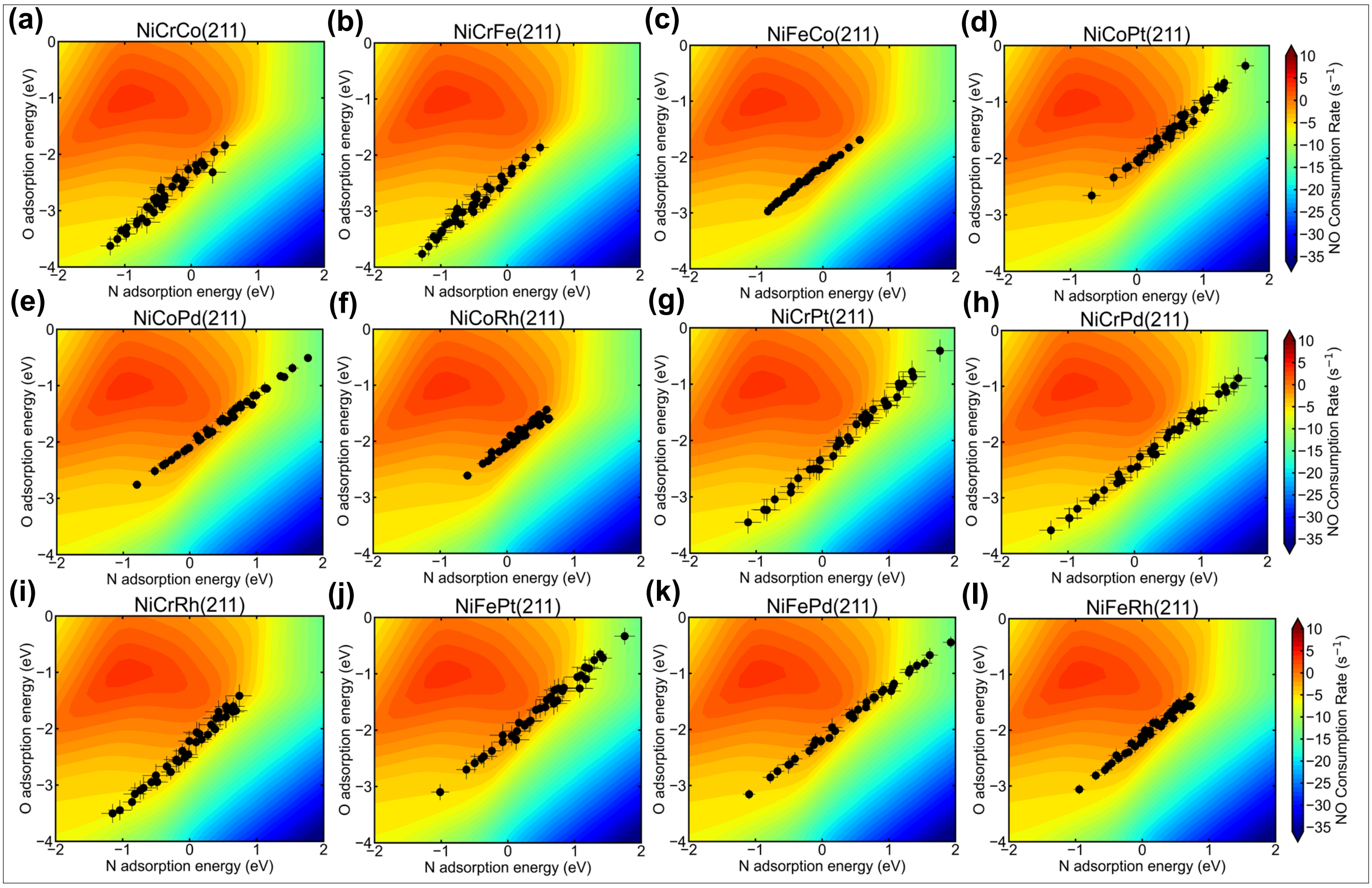


**Figure S19. Ensemble-wise adsorption energies and reaction rates for alloy fcc(211) surfaces:** Ensemble-wise adsorption energies overlaid on the NO consumption activity volcano for fcc(111) surfaces of (a) NiCrCo (b) NiCrFe (c) NiFeCo (d) NiCoPt (e) NiCoPd (f) NiCoRh (g) NiCrPt (h) NiCrPd (i) NiCrRh (j) NiFePt (k) NiFePd and (l) NiFeRh. Error bars indicate corresponding standard deviation. In the figure, fcc is face-centered cubic.

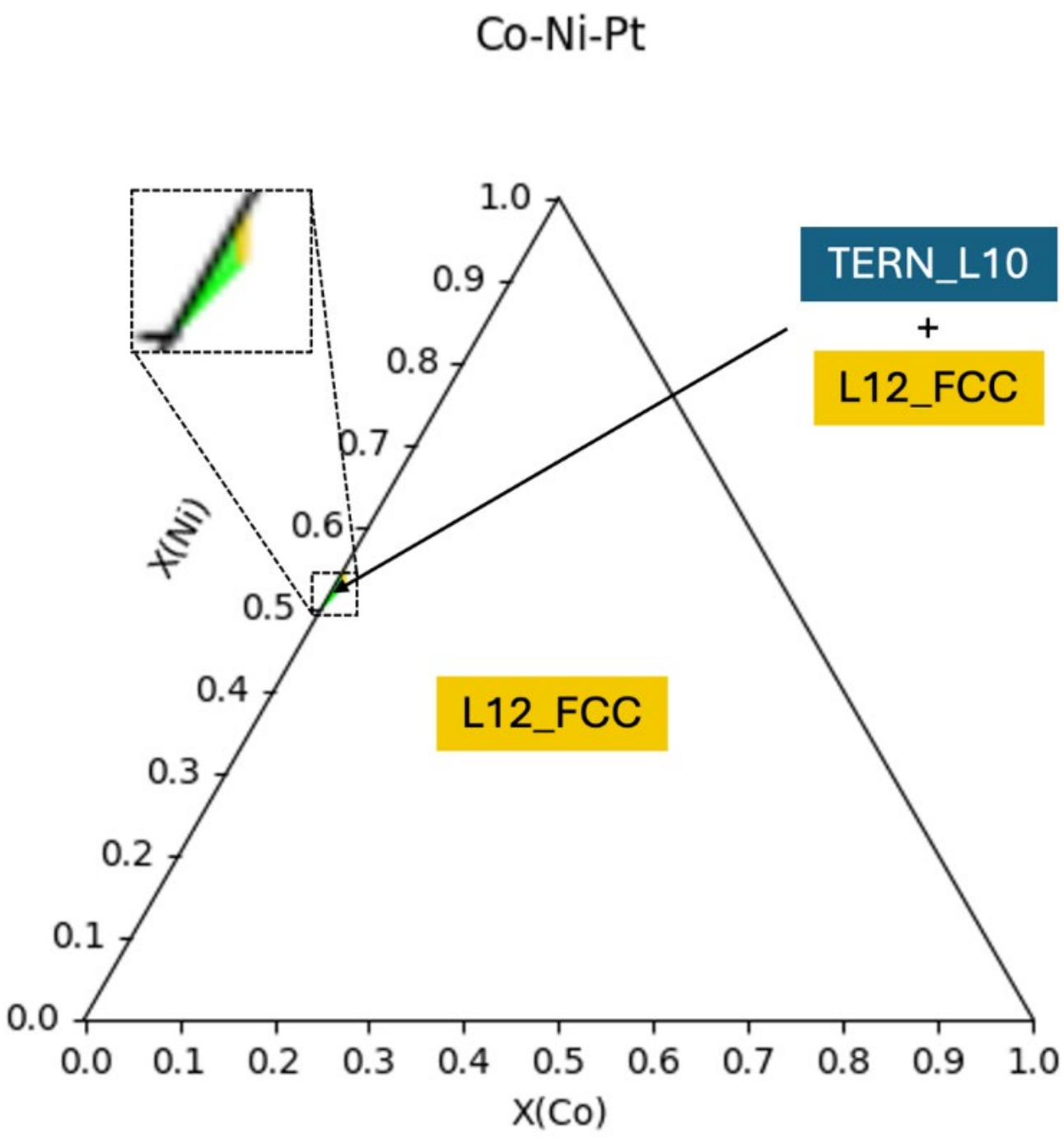


**Figure S20: Simulated ternary phase diagram (Ternary Isotherm Approximation) of Ni-Co-Pt**: Generated using the Calphad software and experimental unary elemental and binary datasets for Ni-Co[92], Ni-Pt [91], and Co-Pt[93] at 900 K. Database can be made available upon request. The ordered FCC L12 (an fcc Gamma Phase) is stable for the predominant compositional space. There is a small 2-phase region between approximately 50-55 at. % Ni : 0-0.1 at. % Co (: balance Pt) of the L10 phase (commonly observed in Fe-Ni and Fe-Pt) and the L12 phase. This phase behavior thus only becomes of interest at negligible compositions of Co, which is likely not within the scope of design space for this work. Furthermore, the 2-phase region disappears entirely at temperatures above approximately 930 K, above which L12 is stable across the entire compositional space of the Co-Ni-Pt ternary. Within the temperature region of interest, i.e., 900-1300 K, melting is not a concern as the regime of melting for this ternary is significantly higher at 1728-2041 K. Therefore, ordered fcc is thermodynamically stable for the temperatures and compositional design space of interest for this work. In the figure, fcc is face-centered cubic, TERN_L10 is the ternary L10 phase, and X indicates mole fractions.

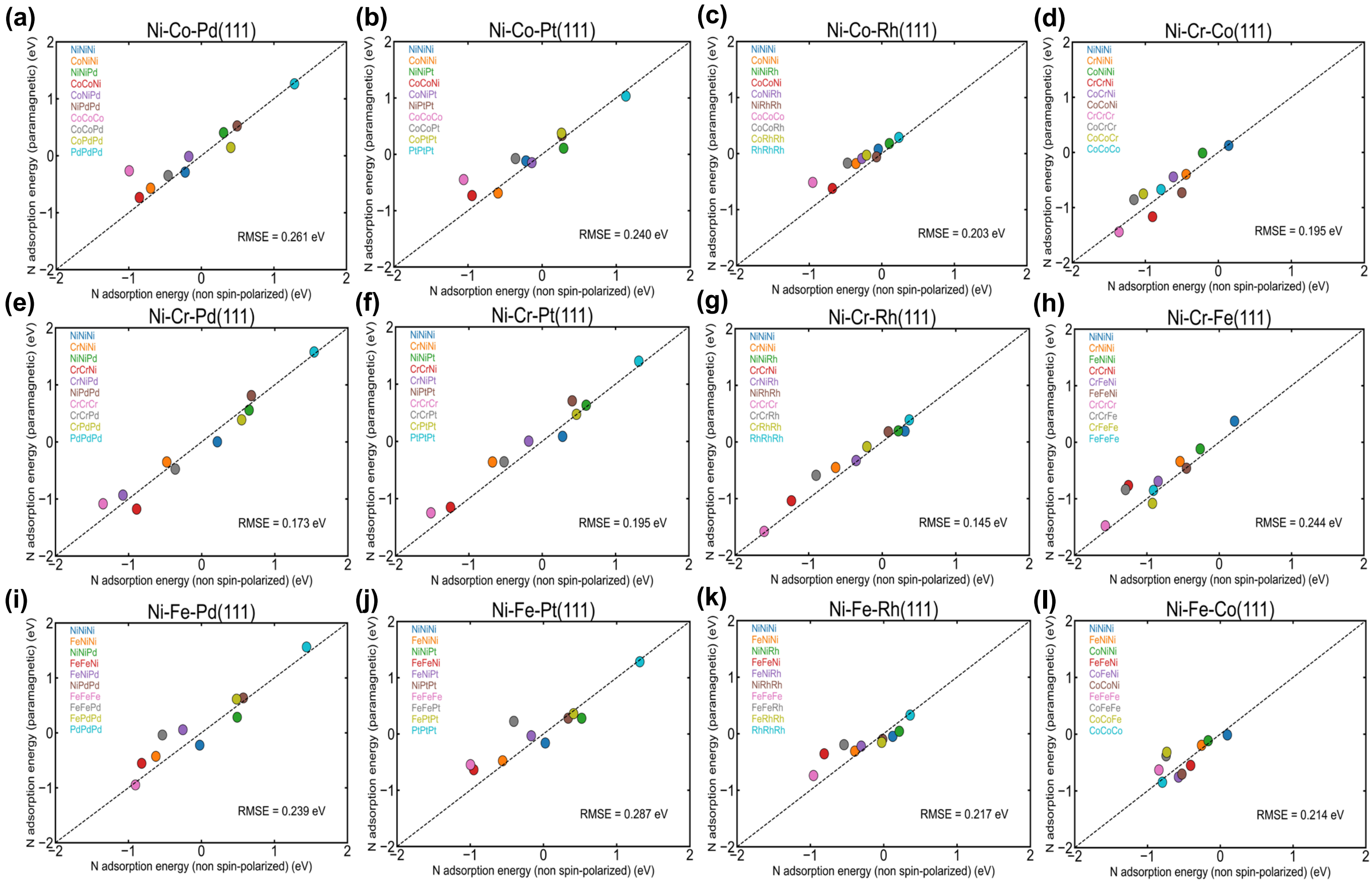


**Figure S21. Comparison of N adsorption energies with/without spin polarization**: Parity plots comparing the adsorption energies of N computed with and without spin-polarization for the fcc(111) surface of (a) NiCoPd (b) NiCoPt (c) NiCoRh (d) NiCrCo (e) NiCrPd (f) NiCrPt (g) NiCrRh (h) NiCrFe (i) NiFePd (j) NiFePt (k)NiFeRh and (l) NiFeCo. Colors indicate the adsorption site ensembles for each alloy. In the figure, fcc is face-centered cubic.

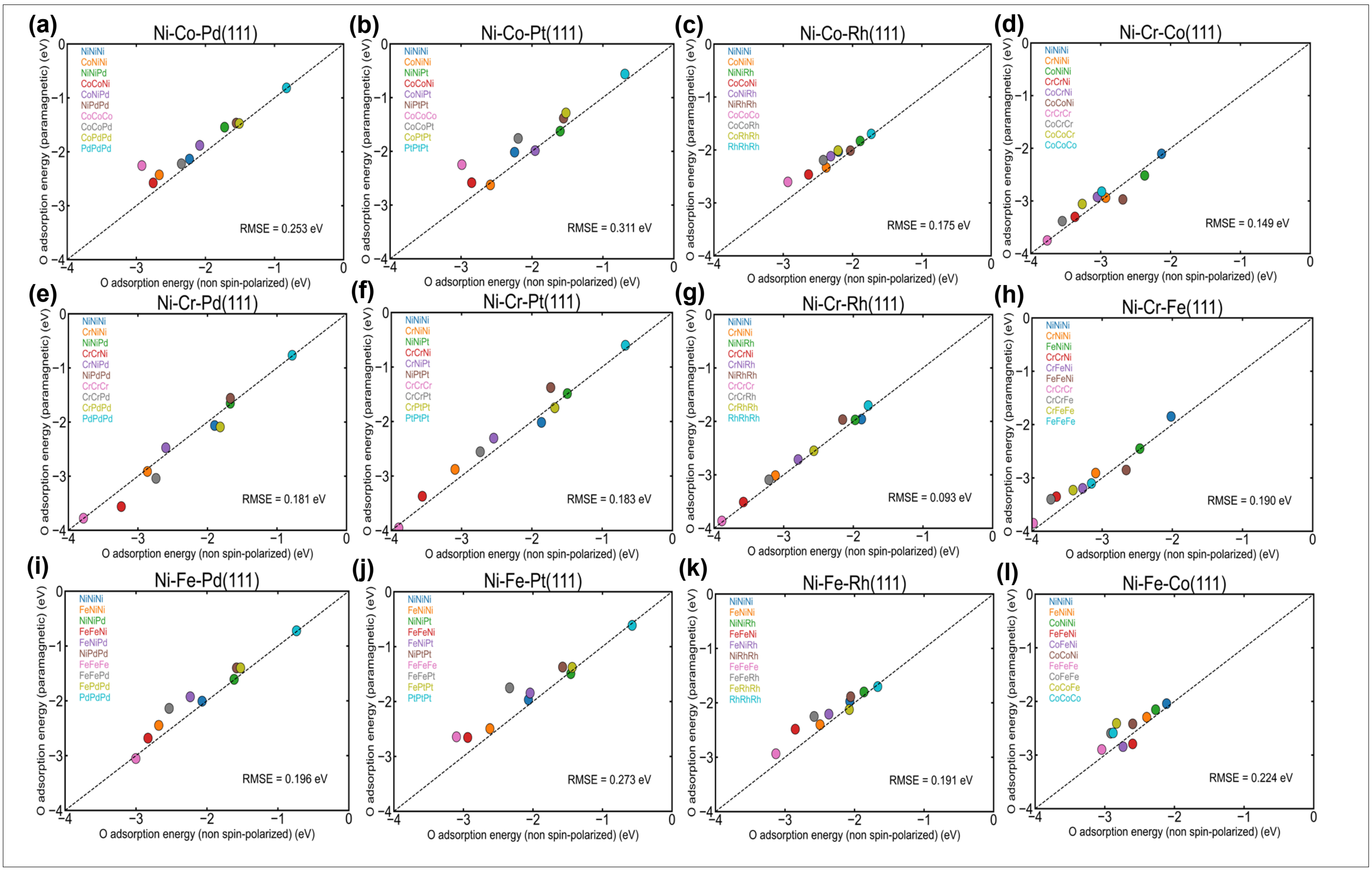


**Figure S22. Comparison of O adsorption energies with/without spin polarization**: Parity plots comparing the adsorption energies of O computed with and without spin-polarization for the fcc(111) surface of (a) NiCoPd (b) NiCoPt (c) NiCoRh (d) NiCrCo (e) NiCrPd (f) NiCrPt (g) NiCrRh (h) NiCrFe (i) NiFePd (j) NiFePt (k)NiFeRh and (l) NiFeCo. Colors indicate the adsorption site ensembles for each alloy. In the figure, fcc is face-centered cubic.

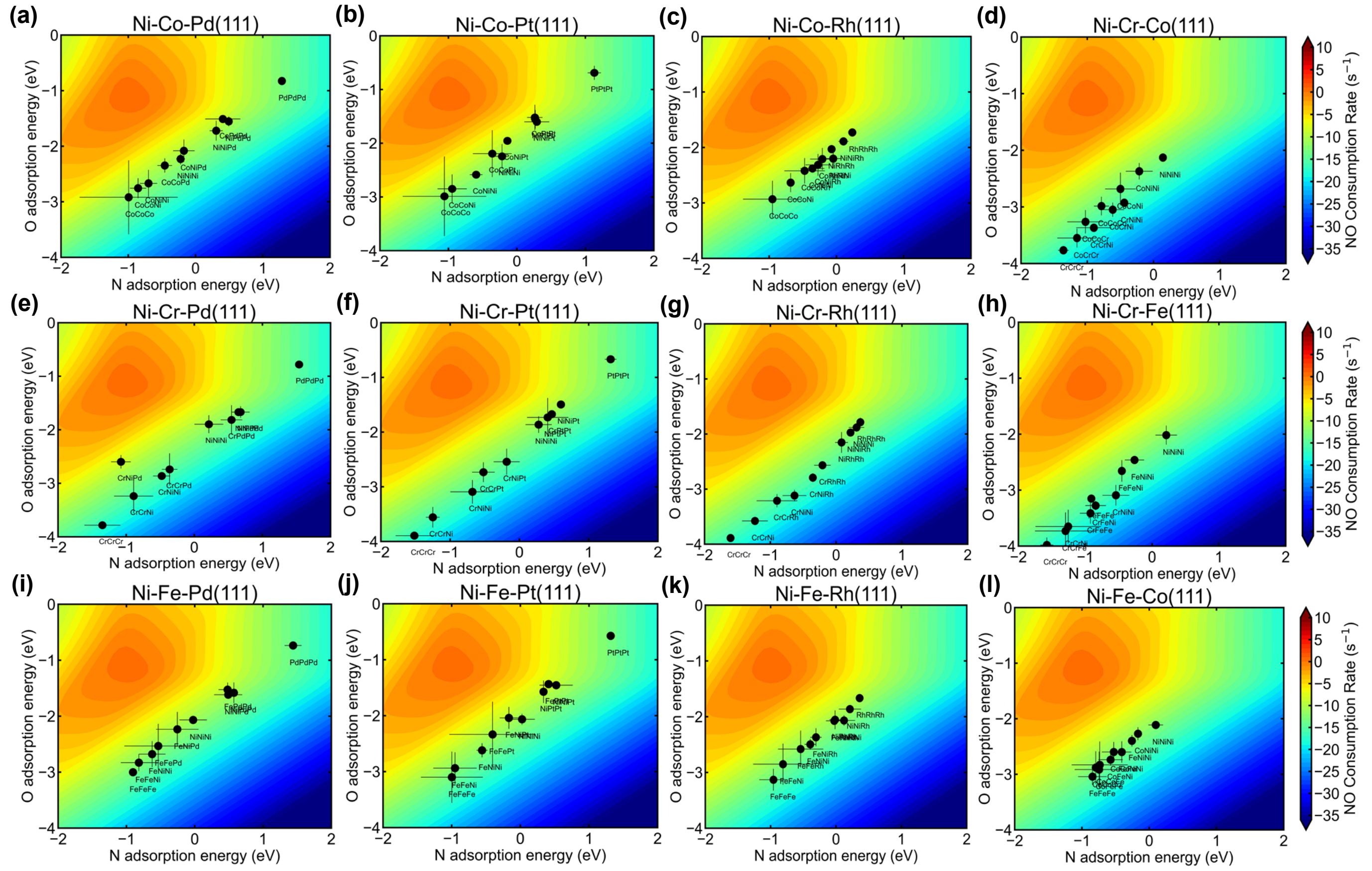


**Figure S23. Ensemble-wise spin-aware adsorption energies and reaction rates for alloy fcc(111) surfaces:** Ensemble-wise adsorption energies overlaid on the NO consumption activity volcano for fcc(111) surfaces of (a) NiCrCo (b) NiCrFe (c) NiFeCo (d) NiCoPt (e) NiCoPd (f) NiCoRh (g) NiCrPt (h) NiCrPd (i) NiCrRh (j) NiFePt (k) NiFePd and (l) NiFeRh. Error bars indicate difference between spin-polarized and non-spin-polarized calculations. In the figure, fcc is face-centered cubic.

**Table S1:** Preferred adsorption site for every adsorbate and metal combination on fcc(111) surfaces

| Metal | C | O | N | CO | NO |
|---|---|---|---|---|---|
| Ag | fcc | fcc | fcc | atop | fcc |
| Au | fcc | fcc | fcc | atop | bridge |
| Co | hcp | fcc | hcp | hcp | hcp |
| Cu | fcc | fcc | fcc | fcc | fcc |
| Ni | hcp | fcc | fcc | fcc | fcc |
| Pd | fcc | fcc | fcc | fcc | fcc |
| Pt | fcc | fcc | fcc | fcc | fcc |
| Re | hcp | hcp | hcp | atop | atop |
| Rh | hcp | fcc | hcp | hcp | hcp |
| Ru | hcp | hcp | hcp | atop | hcp |
| Ir | hcp | fcc | hcp | atop | atop |
| Os | hcp | hcp | hcp | atop | atop |

**Table S2**: Preferred adsorption site for every adsorbate and metal combination on fcc(100) surfaces

| Metal | C | O | N | CO | NO |
|---|---|---|---|---|---|
| Ag | 4-fold hollow | 4-fold hollow | 4-fold hollow | atop | 4-fold hollow |
| Au | 4-fold hollow | bridge | 4-fold hollow | bridge | bridge |
| Co | 4-fold hollow | 4-fold hollow | 4-fold hollow | 4-fold hollow | Bridge |
| Cu | 4-fold hollow | 4-fold hollow | 4-fold hollow | atop | 4-fold hollow |
| Ni | 4-fold hollow | 4-fold hollow | 4-fold hollow | 4-fold hollow | 4-fold hollow |
| Pd | 4-fold hollow | 4-fold hollow | 4-fold hollow | bridge | bridge |
| Pt | 4-fold hollow | bridge | bridge | bridge | bridge |
| Re | 4-fold hollow | bridge | 4-fold hollow | atop | atop |
| Rh | 4-fold hollow | 4-fold hollow | 4-fold hollow | bridge | bridge |
| Ru | 4-fold hollow | 4-fold hollow | 4-fold hollow | atop | bridge |
| Ir | 4-fold hollow | bridge | 4-fold hollow | atop | bridge |
| Os | 4-fold hollow | bridge | 4-fold hollow | atop | atop |

**Table S3**: Preferred adsorption site for every adsorbate and metal combination on fcc(211) surfaces

| Metal | C | O | N | CO | NO |
|---|---|---|---|---|---|
| Ag | 5-fold hollow | 5-fold hollow | 5-fold hollow | step atop | step bridge |
| Au | hollow (a) | step bridge | hollow (a) | step atop | step bridge |
| Co | 5-fold hollow | hollow (a) | 5-fold hollow | step atop | step bridge |
| Cu | 5-fold hollow | hollow (a) | 5-fold hollow | step atop | step bridge |
| Ni | 5-fold hollow | hollow (a) | 5-fold hollow | hollow (a) | hollow (a) |
| Pd | 5-fold hollow | hollow (b) | hollow (a) | step bridge | step bridge |
| Pt | hollow (a) | step bridge | hollow (a) | step atop | step bridge |
| Re | 5-fold hollow | step bridge | hollow (a) | step bridge | step atop |
| Rh | 5-fold hollow | step bridge | hollow (a) | step bridge | step bridge |
| Ru | 5-fold hollow | step bridge | hollow (a) | step atop | step atop |
| Ir | hollow (a) | step bridge | step bridge | step atop | step atop |
| Os | hollow (a) | step bridge | hollow (a) | step atop | step atop |

**Table S4**: Activation barriers and reaction energies for fcc (111) surfaces. Values are reported in eV.

| **Metal** | $O_2 + 2^* \rightarrow 2O^*$ | | $N_2 + 2^* \rightarrow 2N^*$ | | $NO + 2^* \rightarrow N^* + O^*$ | | $CO + 2^* \rightarrow C^* + O^*$ | | $CO_2 + 2^* \rightarrow CO^* + O^*$ | |
|---|---|---|---|---|---|---|---|---|---|---|
| | $\Delta E$ | $E_{TS}$ | $\Delta E$ | $E_{TS}$ | $\Delta E$ | $E_{TS}$ | $\Delta E$ | $E_{TS}$ | $\Delta E$ | $E_{TS}$ |
| Ag | -0.601 | 1.141 | 6.279 | 7.027 | 1.891 | 3.025 | 4.833 | 5.508 | 2.936 | 2.894 |
| Au | 0.218 | 1.861 | 5.57 | 6.728 | 1.946 | 3.159 | 4.166 | 5.089 | 3.218 | 3.355 |
| Co | -5.617 | -1.481 | -1.526 | 1.16 | -4.52 | -1.669 | -1.486 | 0.434 | -1.503 | 0.361 |
| Cu | -2.818 | -0.258 | 3.233 | 4.393 | -0.741 | 0.959 | 2.334 | 3.362 | 1.132 | 1.806 |
| Ni | -4.645 | -1.114 | -0.645 | 1.644 | -3.594 | -1.03 | -0.733 | 1.071 | -1.054 | 0.823 |
| Pd | -2.262 | 0.204 | 1.036 | 3.380 | -1.561 | 0.443 | 0.512 | 2.167 | 0.239 | 1.82 |
| Pt | -1.905 | 0.607 | 1.005 | 3.540 | -1.398 | 0.747 | 0.511 | 2.295 | 0.625 | 2.296 |
| Rh | -3.664 | -0.752 | -0.083 | 2.229 | -2.822 | -0.548 | -0.349 | 1.404 | -0.385 | 0.986 |
| Ir | -3.086 | -0.733 | 0.376 | 2.574 | -2.303 | -0.166 | 0.059 | 1.949 | 0.187 | 1.442 |
| Os | -4.462 | -1.333 | -0.601 | 1.481 | -3.48 | -1.221 | -0.75 | 0.942 | -0.403 | 0.408 |
| Ru | N/A | N/A | -0.836 | 1.198 | -3.851 | -1.523 | -1.009 | 0.686 | -0.886 | 0.312 |
| Re | -5.712 | -1.804 | -1.702 | 0.916 | -4.655 | -1.888 | -1.672 | 0.436 | -1.321 | 0.759 |

**Table S5**: Activation barriers and reaction energies for fcc (100) surfaces. Values are reported in eV.

| **Metal** | $O_2 + 2^* \rightarrow 2O^*$ | | $N_2 + 2^* \rightarrow 2N^*$ | | $NO + 2^* \rightarrow N^* + O^*$ | | $CO + 2^* \rightarrow C^* + O^*$ | | $CO_2 + 2^* \rightarrow CO^* + O^*$ | |
|---|---|---|---|---|---|---|---|---|---|---|
| | $\Delta E$ | $E_{TS}$ | $\Delta E$ | $E_{TS}$ | $\Delta E$ | $E_{TS}$ | $\Delta E$ | $E_{TS}$ | $\Delta E$ | $E_{TS}$ |
| Ag | -1.280 | 0.971 | 5.081 | 6.647 | 3.744 | 5.06 | 0.952 | 2.618 | 2.608 | 2.597 |
| Au | 0.679 | 2.117 | 6.069 | 7.073 | 4.293 | 4.855 | 2.426 | 2.713 | 3.564 | 3.003 |
| Co | -5.815 | -1.110 | -3.034 | 0.995 | -2.977 | -0.814 | -5.373 | -2.223 | -1.672 | -0.195 |
| Cu | -3.424 | -0.193 | 1.548 | 4.017 | 0.854 | 2.538 | -1.886 | 0.439 | 0.851 | 1.536 |
| Ni | -4.824 | -0.584 | -1.993 | 1.924 | -2.172 | -0.032 | -4.357 | -0.506 | -1.083 | 0.241 |
| Pd | -1.821 | 0.692 | 0.897 | 4.135 | -0.171 | 1.384 | -1.410 | 0.319 | 0.706 | 1.206 |
| Pt | -0.949 | 0.990 | 1.898 | 4.128 | 0.501 | 1.497 | -0.474 | 1.162 | 1.346 | 0.902 |
| Rh | -3.844 | -0.567 | -0.871 | 2.310 | -1.619 | 0.136 | -3.306 | -0.862 | -0.548 | 0.315 |
| Ir | -2.799 | -0.172 | -0.028 | 2.509 | -0.86 | 0.429 | -2.362 | -0.176 | 0.246 | 1.734 |
| Os | -4.386 | -0.927 | -1.127 | 1.808 | -1.653 | -0.012 | -3.705 | -0.843 | -0.550 | 0.143 |
| Ru | -5.125 | -1.317 | -1.459 | 1.640 | -2.17 | -0.184 | -4.240 | -1.498 | -1.054 | -0.02 |
| Re | -6.630 | -1.513 | -3.721 | 0.526 | -4.021 | -1.426 | -6.124 | -1.929 | -2.202 | -0.282 |

**Table S6**: Activation barriers and reaction energies for fcc (211) surfaces. Values are reported in eV.

| **Metal** | $O_2 + 2^* \rightarrow 2O^*$ | | $N_2 + 2^* \rightarrow 2N^*$ | | $NO + 2^* \rightarrow N^* + O^*$ | | $CO + 2^* \rightarrow C^* + O^*$ | | $CO_2 + 2^* \rightarrow CO^* + O^*$ | |
|---|---|---|---|---|---|---|---|---|---|---|
| | $\Delta E$ | $E_{TS}$ | $\Delta E$ | $E_{TS}$ | $\Delta E$ | $E_{TS}$ | $\Delta E$ | $E_{TS}$ | $\Delta E$ | $E_{TS}$ |
| Ag | -0.217 | 1.066 | 6.702 | 7.342 | 5.106 | 5.668 | 2.221 | 2.862 | 2.82 | 2.719 |
| Au | 0.632 | 1.408 | 6.309 | 6.615 | 4.657 | 4.912 | 2.373 | 2.792 | 3.248 | 3.27 |
| Co | N/A | N/A | -0.976 | -0.005 | -1.782 | -0.708 | -4.294 | -1.702 | N/A | N/A |
| Cu | -2.412 | -0.534 | 3.566 | 4.644 | 2.122 | 3.135 | -0.578 | 0.572 | 1.169 | 1.413 |
| Ni | -3.905 | -1.011 | 0.108 | 0.965 | -0.876 | 0.293 | -3.087 | -1.944 | -0.488 | 0.743 |
| Pd | N/A | N/A | 2.105 | 2.421 | 0.989 | 1.233 | -1.002 | -0.443 | 0.743 | 1.496 |
| Pt | -1.295 | 0.389 | 1.913 | 2.751 | 1.009 | 1.472 | -0.909 | 0.219 | 1.239 | 2.184 |
| Rh | -3.309 | -0.298 | 0.396 | 1.169 | -0.397 | 0.252 | -2.607 | -1.436 | 0.03 | 0.946 |
| Ir | -2.909 | 0.009 | 0.576 | 1.482 | -0.276 | 0.433 | N/A | N/A | 0.566 | 1.493 |
| Os | -4.607 | -1.092 | -0.39 | 0.663 | -1.333 | -0.192 | N/A | N/A | -0.193 | 0.773 |
| Ru | N/A | N/A | -0.422 | 0.671 | -1.268 | -0.215 | -3.731 | -2.081 | -0.543 | 0.422 |
| Re | -6.173 | -1.739 | -1.715 | -0.261 | -2.559 | -1.236 | N/A | N/A | -1.051 | -1.216 |
